# Genome-Wide Analysis of Gene Expression during Early *Arabidopsis* Flower Development

**DOI:** 10.1371/journal.pgen.0020117

**Published:** 2006-07-28

**Authors:** Frank Wellmer, Márcio Alves-Ferreira, Annick Dubois, José Luis Riechmann, Elliot M Meyerowitz

**Affiliations:** Division of Biology, California Institute of Technology, Pasadena, California, United States of America; The Salk Institute for Biological Studies, United States of America

## Abstract

Detailed information about stage-specific changes in gene expression is crucial for the understanding of the gene regulatory networks underlying development. Here, we describe the global gene expression dynamics during early flower development, a key process in the life cycle of a plant, during which floral patterning and the specification of floral organs is established. We used a novel floral induction system in *Arabidopsis,* which allows the isolation of a large number of synchronized floral buds, in conjunction with whole-genome microarray analysis to identify genes with differential expression at distinct stages of flower development. We found that the onset of flower formation is characterized by a massive downregulation of genes in incipient floral primordia, which is followed by a predominance of gene activation during the differentiation of floral organs. Among the genes we identified as differentially expressed in the experiment, we detected a significant enrichment of closely related members of gene families. The expression profiles of these related genes were often highly correlated, indicating similar temporal expression patterns. Moreover, we found that the majority of these genes is specifically up-regulated during certain developmental stages. Because co-expressed members of gene families in *Arabidopsis* frequently act in a redundant manner, these results suggest a high degree of functional redundancy during early flower development, but also that its extent may vary in a stage-specific manner.

## Synopsis

The development of flowers is one of the characteristic features of higher plants. In an effort to gain detailed insights into the molecular processes underlying flower development, the authors have analyzed the expression of the genes of the small plant *Arabidopsis thaliana,* which is widely used by biologists for the study of plant development, during the early stages of flower formation. To this end, they used DNA microarray analysis, a technology that allows the simultaneous detection of thousands of gene transcripts in a single experiment. Because young floral buds of *Arabidopsis* are minute and are difficult to dissect, the authors established a system that allows the simultaneous induction of a large number of flowers on a single plant. Using this system, they identified groups of genes, many of them novel or uncharacterized, that are highly active during distinct stages of flower development. These genes are likely involved in controlling the various developmental changes that take place during the formation of flowers. The authors also found that many of these genes are closely related in sequence, suggesting that they might be involved in similar or identical processes, and thus uncovering a large degree of potential functional redundancy during flower development.

## Introduction

**Figure 1:**
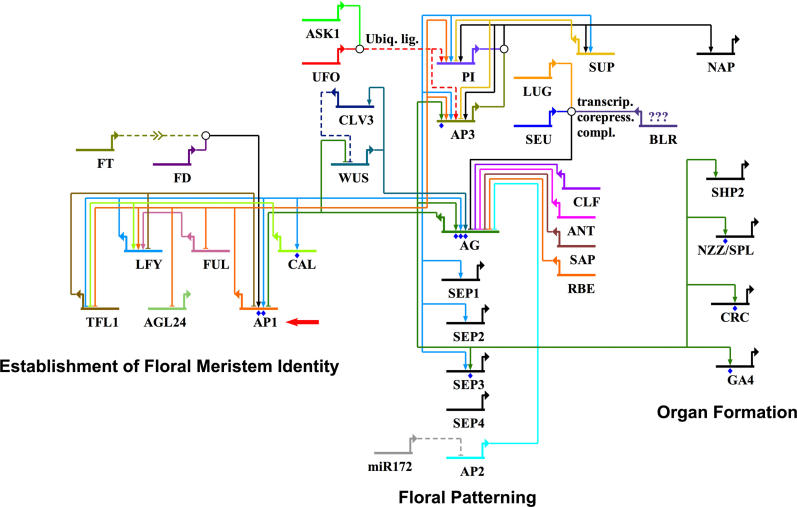
Gene Regulatory Network Controlling Early Flower Development Genes involved in the establishment of floral meristem identity, floral patterning, or floral organ formation, and their regulatory interactions are shown. Certain floral regulators were not included in the diagram, because their positions in the gene regulatory network relative to the genes shown are currently not well understood. Individual genes are represented by horizontal lines with bent arrows and gene symbols (see Table S11 for full gene names). For each gene, upstream inputs and downstream targets are indicated. Activators are connected to their targets by arrows, repressors by blunted lines. Blue dots underneath gene symbols indicate that direct binding to these genes has been demonstrated by chromatin immunoprecipitation or by a combination of in vitro binding studies and in vivo binding site disruptions. Note their small number in the diagram, indicating the limited availability of binding data. White circles represent protein complexes. ASK1 and UFO are part of an ubiquitin ligase complex (“Ubiq. lig.”). SEU, LEU, and perhaps BLR (question marks indicate that a direct interaction of BLR to SEU and/or LEU has not yet been demonstrated) are part of a transcriptional co-repressor complex (“transcrip. corepress. compl.”) controlling AG expression. Protein interactions between MADS-box transcription factors [41] are not depicted (with the exception of AP3 and PI, which are thought to act as an oligate heterodimer [58]), to simplify the diagram. Arrows for FT symbolize a long-range transport of FT mRNA from leaves to shoot apices [59]. Dashed lines indicate that gene products do not function as transcriptional regulators. A red arrow marks the position of AP1 in the gene regulatory network. Diagram was generated using BioTapestry [60] and is based on published data (see Table S11 for selected references).

Over the past two decades, flower development has attracted widespread attention as an excellent model system for studying organogenesis in plants at a molecular level [1]. Extensive genetic analyses, especially in the model plant Arabidopsis thaliana and in Antirrhinum majus have led to the identification of several key regulatory genes of this important biological process, and the regulatory interactions between these genes have been, in many cases, elucidated through genetic and molecular analysis [2–4]. The vast majority of the floral regulatory genes identified to date encode transcription factors or other proteins involved in the regulation of gene expression, indicating the existence of a complex gene regulatory network that underlies flower development (Figure 1). Most of these genes act during the very early steps of flower formation, in processes such as the establishment of floral meristem identity, or in the patterning of the floral meristem into distinct domains that give rise to the different types of floral organs (i.e. sepals, petals, stamens, and carpels) [2–4] (Figure 1). In contrast, comparatively few genes have been identified through genetic analysis that function specifically at later stages of flower development, and that control floral organ formation. One possible reason for why these genes might have been missed in genetic screens is that their loss of function might result in subtle phenotypes, so that the corresponding mutant plants are easily missed, or they are excluded from further analysis because of the concomitant isolation of (potentially more interesting) mutants with more severe phenotypic alterations.

Another explanation is based on the fact that in plants, as well as in other organisms, the disruption of a single gene often results in no discernable mutant phenotype, because the loss of its activity can be readily compensated by other genes that control the affected developmental process in an, at least partially, redundant manner. Functional redundancy is frequently mediated by closely related genes that have originated from gene (or genome) duplications and that have retained similar or identical functions [5]. Compared to other eukaryotes, plant genomes are strongly enriched for duplicated genes because of frequent segmental duplications and polyploidization events [6], suggesting a high potential for functional redundancy. However, it is thought that such gene duplicates functionally diverge over time. In fact, it appears that one of the duplicates is frequently lost (indicating that its retention is not beneficial for the organism) or that it may acquire novel functions that were not mediated by the corresponding progenitor gene. Alternatively, disruption of certain regulatory elements in the promoters of the duplicated genes may lead to altered expression patterns and hence to sub-functionalization [6].

In addition to duplicated genes, functional redundancy can also originate from unrelated genes or pathways that are part of buffering mechanisms that protect regulatory networks from the effects of perturbations caused by random mutations [5]. Although there are several examples for genes acting in a redundant manner during flower development [7], the full extent of redundant gene activities in the formation of flowers is currently unknown.

The invention of DNA microarray technology has opened the possibility to study gene expression during development on a genome-wide scale, and rapid advances are being made toward the understanding of the transcriptional programs of several model organisms [8,9], including *Arabidopsis* [10–12]. Several recent studies have aimed at the characterization of the *Arabidopsis* floral transcriptome [11,13–17], leading to the identification of novel flower-expressed genes and in some cases to first insights in how the expression of these genes is controlled by known floral regulators. However, these studies provided only limited information about where and when genes are expressed during flower development. As detailed knowledge about spatio-temporal gene expression is pivotal for a comprehensive understanding of development [8,9], new experimental approaches are needed to improve the resolution of the available expression data, and to obtain a more comprehensive view of the developmental mechanisms and the genes that control flower formation.

The analysis of gene expression during flower development has been hampered mainly by difficulties in isolating sufficient amounts of tissue from distinct floral stages for microarray analysis. This problem is especially pronounced for early flower development in *Arabidopsis*, because floral primordia are minute and are initiated successively so that only one floral bud in an inflorescence is at a given developmental stage [18]. Also, young floral buds are hidden by older, more mature flowers, representing an additional challenge for their dissection.

Here, we describe a novel floral induction system that allows the induction of a large number of synchronized floral buds on a single plant and thus, enables the collection of floral bud populations of distinct developmental stages. We have used this system to analyze gene expression during early flower development on a genome-wide scale by microarray analysis and have identified genes with significant expression changes at different stages of flower development. Among these genes we found a significant enrichment of genes with putative regulatory functions, most of which have not yet been identified through genetic approaches. We also found significant differences in the representation of transcripts from closely related genes at different floral stages, suggesting varying degrees of functional redundancy during distinct stages of flower development.

## Results/Discussion

### Induction of Synchronized Flower Development

**Figure 2:**
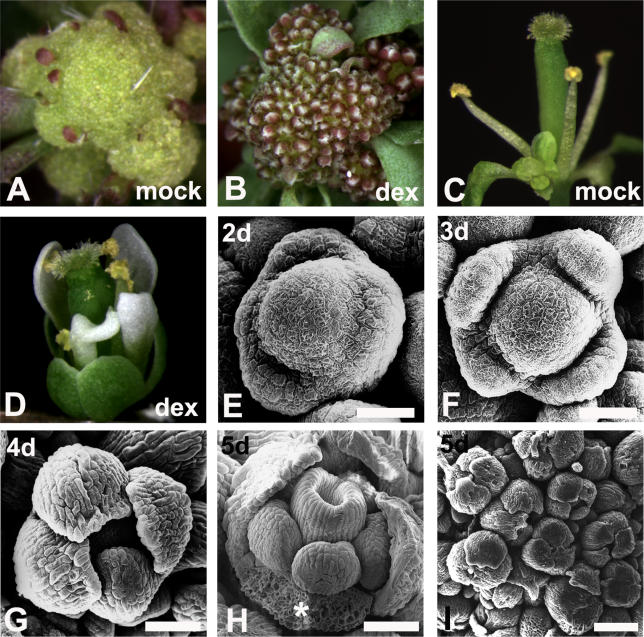
Floral Induction in *ap1 cal* Double Mutant Plants by AP1-GR Activation While no phenotypic response was observed in mock-treated (mock) plants (A), the activation of AP1-GR in the inflorescences of *ap1 cal* plants by dexamethasone (dex) treatment led to a massive induction of floral primordia (B). Images were taken 6 d after a single dexamethasone treatment. (C–D) Fertile flowers of 35S:AP1-GR *ap1 cal* plants 4 wk after treatment with a mock solution (C) and 13 d after a single dexamethasone treatment (D). AP1-GR activation restores the organ identity defects of *ap1 cal* mutant flowers. (E–I) Scanning electron micrographs of floral buds of 35S:AP1-GR *ap1 cal* plants at different time points (as indicated) after a single dexamethasone treatment. In (H), an asterisk indicates the position of a sepal that was removed for a better visibility of the inner whorl organs. Scale bars: 20 μm in (E) to (H) and 100 μm in (I).

The system for the induction of synchronized floral development (Figure 2) is based on plants with loss-of-function mutations in the closely related genes *APETALA1 (AP1)* and *CAULIFLOWER (CAL),* which regulate the initiation of flower development in a redundant manner [19,20]. Flower formation in *ap1 cal* double mutants is (temporarily) blocked and instead, these plants undergo a massive over-proliferation of inflorescence-like meristems, leading to a cauliflower-like appearance (Figure 2A). *ap1 cal* plants eventually flower after a long delay compared to wild-type plants, but the flowers are abnormal and lack sepals and petals (Figure 2C). Because ectopic expression of AP1 in wild-type plants causes the transformation of vegetative and inflorescence meristems into floral meristems [21] (Figure S1), we reasoned that a specific activation of AP1 in the inflorescence-like meristems of *ap1 cal* double mutants might lead to their simultaneous transformation and subsequently to synchronous flower development. To test this, we generated transgenic *ap1 cal* plants expressing a fusion protein of AP1 and the hormone-binding domain of the rat glucocorticoid receptor (GR) from the constitutive cauliflower mosaic virus 35S promoter. Treatment of 35S:AP1-GR *ap1 cal* inflorescences with the synthetic steroid hormone dexamethasone, which activates the AP1-GR fusion protein [22], led to a massive formation of floral buds (Figure 2B), whereas mock-treated control plants showed no phenotypic response (Figure 2A). Examination of the floral buds at different time points after the dexamethasone treatment revealed that they closely resemble those of wild-type plants (as described in [18]), both morphologically and with respect to their temporal progression through the different stages of flower development (Figure 2E–2H). In addition, the hundreds of floral buds that were produced on a single plant were relatively synchronized (Figure 2I), at least until day 5 after the treatment, when most buds had reached stage 7 of flower development. At this stage, all floral organs have been initiated and are undergoing rapid differentiation. After day 5, synchronization was gradually lost (unpublished data), possibly due to space constraints within the compact inflorescence. In contrast to flowers eventually generated by mock-treated 35S:AP1-GR *ap1 cal* plants after a long delay (Figure 2C), mature flowers of dexamethasone-treated plants had formed sepals and petals (Figure 2D) and resembled wild-type flowers (although certain developmental defects, e.g. narrow petals, petalloid sepals, or a reduction in the number of floral organs were observed as well). Thus, activation of AP1-GR by a single dexamethasone treatment not only induced synchronous flower development but was also sufficient to rescue the organ identity defects of *ap1 cal* mutant flowers.

The floral induction system presented here has several conceptual and experimental advantages compared to a previously described approach that allows the induction of synchronized reproductive organ development by specific activation of the floral homeotic factor AGAMOUS (AG) in an *ap1 cal* background [14]: i) Activation of AP1 leads to the formation of flowers with all four types of floral organs and not only to that of stamens and carpels, as in the case of AG, allowing the analysis of all aspects of early flower development; ii) Only a subset of plants exhibit a phenotypic response to AG activation, while all AP1-GR *ap1 cal* plants treated with dexamethasone initiate flower development; iii) In the AP1-GR based system, the blockage of flower development that occurs in *ap1 cal* double mutants is released by simply compensating for the loss of endogenous AP1/CAL activity. In contrast, endogenous *AG* is not expressed during the earliest stages of flower formation [15,23], and the mechanism by which ectopic AG activity is able to override the normal requirement for AP1/CAL during the initiation of flower development is currently unknown.

### Global Analysis of Stage-Specific Gene Expression

**Figure 3:**
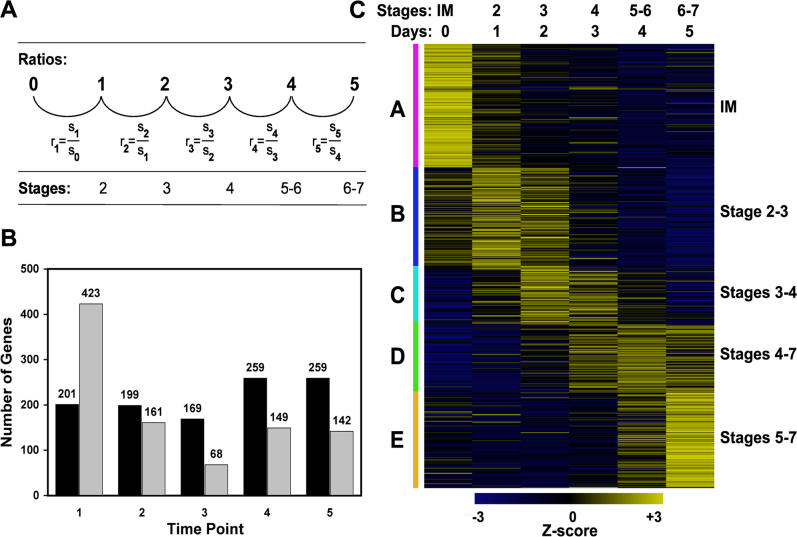
Experimental Design and Results (A) Calculation of expression ratios. RNA samples from tissues collected on two consecutive days were co-hybridized to microarrays. Thus, ratios r_1_ to r_5_ are direct experimental ratios that were calculated for each gene represented on the array using its normalized signal intensities (s) at the individual time points. (B) Summary of gene expression changes observed in the experiment. The number of genes that were up- or down-regulated 1, 2, 3, 4, or 5 days after AP1-GR activation relative to the previous time point is indicated. Black bars represent up-regulated and gray bars down-regulated genes. (C) Heat map representing Z-score normalized signal intensities of 1,653 genes showing significant expression changes in the experiment (as derived from ratios r_1_-r_5_). Yellow indicates high, and blue indicates low expression. Genes were clustered into five groups (A–E; indicated on the left) with predominant expression during certain stages of early flower development (indicated on the right). The approximate developmental stage of the floral buds at the different time points is indicated in (A) and (C). IM: inflorescence-like meristem.

**Figure 4:**
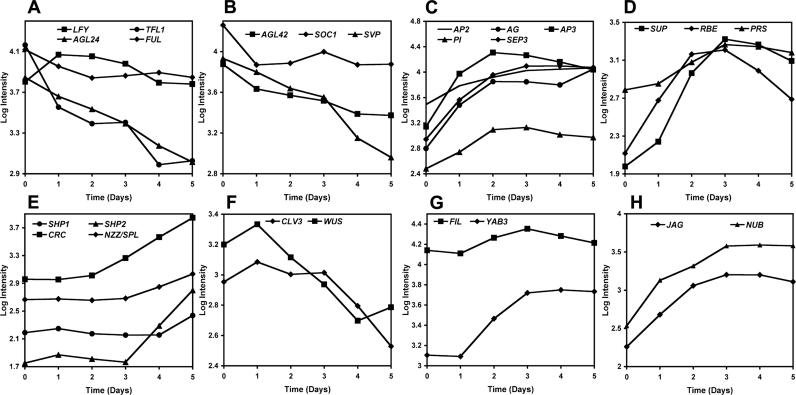
Microarray Results for Selected Floral Regulatory Genes Log_10_-transformed signal intensities at the individual time points of the experiment are shown. (A) Expression dynamics of previously identified AP1 response genes that regulate the initiation of flower formation (see Figure 1). The floral meristem identity gene *LEAFY (LFY)* is rapidly upregulated upon AP1-GR activation, whereas *TERMINAL FLOWER1 (TFL1)* and *AGAMOUS-LIKE 24 (AGL24)* are repressed. The slight reduction in expression of *FRUITFULL (FUL)* was not judged statistically significant in our analysis. (B) Repression of *AGAMOUS-LIKE42 (AGL42), SHORT VEGETATIVE PHASE (SVP),* and *SUPPRESSOR OF OVEREXPRESSION OF CO 1 (SOC1).* (C) Expression dynamics of genes involved in specifying the identity of floral organs. *AP3*: *APETALA3*; *PI*: *PISTILLATA*; *AG*: *AGAMOUS*; *SEP3*: *SEPALLATA3*; *AP2*: *APETALA2*. (D) Activation of genes involved in floral patterning *(SUP: SUPERMAN)* or organ primordia formation *(RBE: RABBIT EARS; PRS: PRESSED FLOWERS).* (E) Induction of genes involved in carpel or stamen primordia development was detected towards the end of the time course experiment *(SHP1*: *SHATTERPROOF1*; *SHP2*: *SHATTERPROOF2*; *CRC*: *CRABS CLAW*; *NZZ/SPL*: *NOZZLE/SPOROCYTELESS).* Expression of *SHP1* was not judged significantly changed in the experiment in contrast to that of its paralog *SHP2*. This result is in agreement with the reported induction of *SHP2* at stage 6, one stage earlier than that of *SHP1* [61,62]. (F) The meristem regulatory genes *WUSCHEL (WUS)* and *CLAVATA3 (CLV3)* were gradually downregulated during the course of the experiment. The increase in *WUS* expression on day 5 likely marks the onset of its expression in stamen primordia [16]. (G) Similar expression profiles (correlation coefficient of 0.84) were observed for *FILAMENTOUS FLOWER (FIL)* and *YABBY3 (YAB3)* in agreement with their largely identical expression patterns in developing flowers [29]. (H) Co-expression of *JAGGED (JAG)* and its paralog *NUBBIN (NUB).* Gene identifiers and references are listed in Table S1.

We used the 35S:AP1-GR *ap1 cal* floral induction system to analyze gene expression during early flower development on a genome-wide scale (Figure 3). To this end, we treated inflorescences with dexamethasone and then collected tissue immediately after the treatment, as well as at 1-d intervals for the following 5 d. For the detection of stage-specific changes in gene expression, RNA derived from consecutive time points was co-hybridized to microarrays representing ~26,700 *Arabidopsis* genes (Figure 3A). Most of the previously characterized floral regulators (Table S1) were among the 1,653 genes that were detected as differentially expressed in this experiment (Table S2). We compared the microarray results for these genes (Figure 4) to their published expression patterns and found them, in general, to be in good agreement. For example, up-regulation of the floral homeotic genes *AG, APETALA3 (AP3),* and *PISTILLATA (PI),* which are involved in specifying floral organ identity [2,3,4] (Figure 1), was detected within the first 2 d after AP1-GR activation and subsequently, their expression levels remained high throughout the rest of the experiment (Figure 4C). These expression profiles are in agreement with the reported induction of these genes in floral meristems at stage 3 and their continued expression in developing floral organs [23–25]. We also found a moderate upregulation of the floral homeotic gene *APETALA2 (AP2)* (Figure 4C), which is broadly expressed in inflorescence meristems and developing floral buds [26]. Thus, AP1 activity is not required to induce *AP2* expression but appears to promote *AP2* expression during early flower development.

Expression of the meristem regulatory genes *WUSCHEL (WUS)* and *CLAVATA3 (CLV3)* was gradually reduced during the course of the experiment (Figure 4F) in accordance with the progressive decrease, and eventual termination, of meristematic activity in developing flowers [27,28]. In contrast, expression levels of *SHATTERPROOF 1* and *2 (SHP1/2), CRABS CLAW (CRC),* and *NOZZLE/SPOROCYTELESS (NZZ/SPL),* which are involved in the development of the reproductive floral organs, remained unchanged during the first few days of the experiments, but started to increase after day 3 when stamen and carpel primordia were initiated (Figure 4E). Furthermore, the expression profiles of *FILAMENTOUS FLOWER (FIL)* and *YABBY3 (YAB3)* (Figure 4G), for which largely identical expression patterns in flowers have been reported [29], were highly correlated, indicating that co-expression of genes was reliably detected by the microarray analysis. We also detected a simultaneous and rapid upregulation of *JAGGED (JAG)* and *NUBBIN (NUB)* (Figure 4H), two closely related C2H2 zinc-finger protein-coding genes that act (in a partially redundant manner) in the control of floral organ differentiation [30–32]. Results of in situ hybridizations have shown that expression of *JAG* commences in late-stage 2 flowers [30,31] and that *NUB* is expressed from stage 5 onward [32]. Our microarray data suggest, however, that *NUB* expression is initiated significantly earlier in flower development and temporally parallels that of *JAG*. This discrepancy could be a result of non-localized *NUB* expression in very young floral buds, which would be difficult to detect by in situ hybridizations. A similar idea has been put forward for inconsistencies between the reported expression pattern of *CRC* and its gene expression profile as determined by microarray analysis [17].

We also found that the expression profiles of genes previously identified as being regulated by AP1 during the establishment of floral meristem identity (Figure 1) changed rapidly after activation of the AP1-GR fusion protein. Expression of the floral meristem identity regulator *LEAFY (LFY)* was up-regulated, whereas expression of the shoot-identity gene *TERMINAL FLOWER 1 (TFL1)* and of *AGAMOUS-LIKE 24 (AGL24)* (a regulator of inflorescence fate and a putative direct target of AP1 [22]) were strongly repressed (Figure 4A). In contrast, we observed only a weak effect of AP1 activity on the expression of *FRUITFULL (FUL)* (Figure 4A), which acts redundantly with *AP1* and *CAL* during the establishment of floral meristem identity [20]. *FUL* is expressed ectopically in meristems of *ap1* and *ap1 cal* mutant plants, suggesting that AP1 is a repressor of *FUL* [20]. Other genes with known or presumed roles in flower development whose expression responded rapidly to AP1-GR activation included the MADS-box transcription factor-coding genes *SUPPRESSOR OF CO-OVEREXPRESSION 1 (SOC1)*, the *SOC1* paralog *AGAMOUS-LIKE 42 (AGL42),* and *SHORT VEGETATIVE PHASE (SVP)* (Figure 4B), all of which were repressed. It is noteworthy that the repression of the floral pathway integrator *SOC1* by AP1 is in agreement with the down-regulation of *SOC1* expression in stage 1 floral buds [33] when AP1 becomes active. Further experimentation will be required to determine whether this interaction is direct or indirect.

Taken together, the results of our analysis validate the microarray data, and show that the development of the floral buds induced by AP1-GR activation closely resembles that of wild-type flowers, not only at the morphological but also at the molecular level.

**Figure 5:**
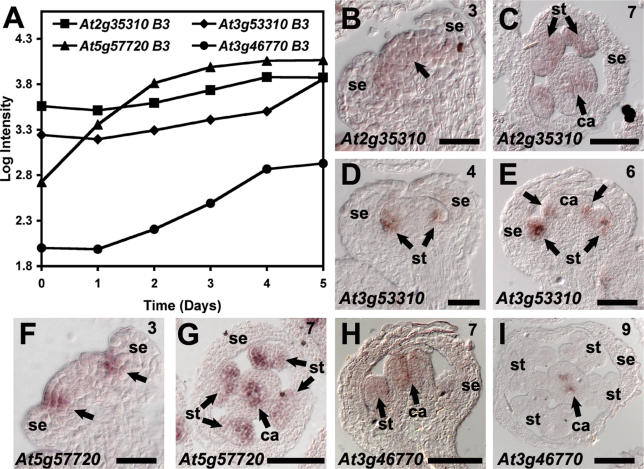
Expression Patterns of Four Genes Encoding B3-Domain-Containing Proteins in Early-Stage Wild-Type Flowers (A) Microarray results: log_10_-transformed signal intensities at the individual time points are shown for the genes tested. (B–I) Results of *in situ* hybridizations. Expression patterns were analyzed in early-stage flowers of wild-type plants. Arrows point to regions of expression. Expression of *At2g35310* was first detected throughout the center of young floral buds (B). At later stages, expression was confined to stamen and carpel primordia (C). Expression of *At3g53310* was first detected at stage 4 throughout very young stamen primordia (D). At stage 6, expression was observed in stamen, as well as in carpel primordia (E). Expression of *At5g57720* was first detected in stage 3 floral buds adjacent to the emerging sepal primordia (F). Its expression at later stages resembled that of *At2g35310* (compare panels [G and C]). Weak expression of *At3g46770* in stamen and carpel primordia was first observed in stage 7 floral buds (H). At later stages, expression was confined to the margins of the central septum of the gynoecium (I). (C, G, and I) show transverse sections; in all other panels, longitudinal sections are shown. Numbers indicate approximate floral stages. Scale bars: 30 μm (B, D, and F); 50 μm (E); 100 μm in all others. ca, carpel; se, sepal; st, stamen.

**Figure 6:**
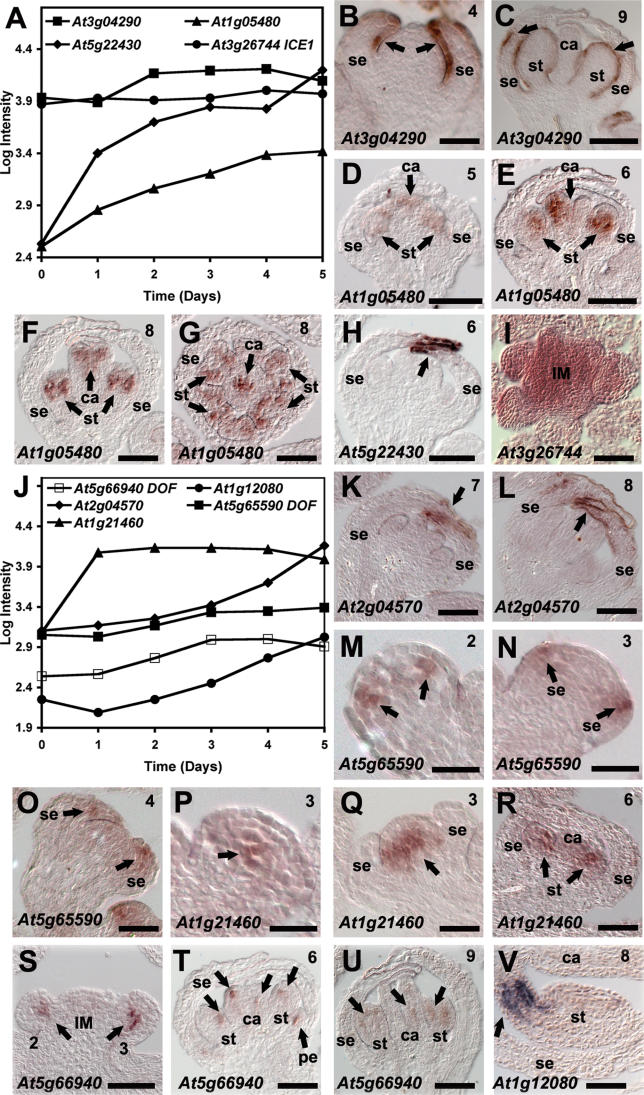
Results of In Situ Hybridizations for Selected Genes Expression patterns were analyzed in early-stage wild-type flowers. Arrows point to regions of expression. (A and J) Log_10_-transformed signal intensities at the individual time points are shown for the genes tested. (B) Expression of *At3g04290,* which encodes a lipase, was first detected at stage 4 in the epidermis of emerging sepals. At later stages, expression was also observed in the epidermis of stamens (C). (D to G) Expression of *At1g05480,* which encodes a SNF2-domain containing protein, was detected in developing stamens and carpels. (H) Expression of *At5g22430,* which encodes a protein of unknown function, was first detected in the tip of sepals around stage 6 of flower development. (I) Expression of *At3g26744,* encoding the bHLH transcription factor *INDUCER OF CBF EXPRESSION 1,* was found in the inflorescence meristem and throughout developing flowers. (K and L) Expression of *At2g04570*, encoding a lipase related to *At3g04290* (see above), was first detected in stage 7 floral buds in the epidermis of sepals and, in contrast to *At3g04290,* appeared to be confined to sepals also at later stages of flower development (compare L and C). (M–O) Expression of *At5g65590,* which encodes a Dof-type zinc-finger protein, was first observed in a small number of cells in stage 2 floral meristems (M). At stage 3 (N), expression was detected in incipient sepal primordia, and expression appeared to continue exclusively in developing sepals at later stages of development. (P–R) Expression of *At1g21460,* encoding a nodulin MtN3 family protein, was found at early stage 3 in a few cells in the center of the floral apex (P). At late stage 3, the expression domain of this gene was significantly enlarged (Q). No signal was found in the upper cell layers of the meristem. At later stages, expression was detected in stamen primordia (R). (S–U) Expression of *At5g66940,* which encodes a Dof-type zinc-finger containing protein, was observed in a small number of cells in very young floral buds (S), as well as in patchy pattern in young floral organ primordia (T). At later stages, its expression was confined to stamens and carpels (U). (V) Expression of *At1g12080,* which encodes a protein of unknown function, was first detected in a region at the base of stamen primordia, which gives rise to the filament. (G) and (I) are transverse sections; in all others longitudinal sections are shown. Numbers indicate approximate floral stages. Scale bars: 30 μm (B and M–Q); 100 μm (C, I, S, U, and V); 50 μm in all other panels. ca, carpel; IM, inflorescence meristem; pe, petal; se, sepal; st, stamen.

We next investigated whether our results could be used to accurately predict the expression dynamics of genes not previously characterized. To this end, we determined by in situ hybridization the expression patterns of several of the differentially expressed genes in wild-type flowers (Figures 5 and 6). For this analysis, we focused on genes that were up-regulated in response to AP1-GR activation. We found that the expression patterns of the genes tested were, in general, in good agreement with our microarray data. For example, several closely related members of the plant-specific family of B3 domain proteins were detected as induced at different time points after AP1-GR activation, suggesting that they might have distinct expression patterns during early flower development. In fact, when we analyzed in detail the expression of four of these genes (Figure 5B–5I), we found that they all are expressed in developing stamens and carpels, as previously predicted [14], but that their temporal and spatial expression shows only partial overlap. For instance, expression of *At5g57720* was first detected in strips adjacent to the emerging sepals (likely marking the cells that give rise to stamen primordia) at stage 3 of flower development (Figure 5F), whereas expression of *At3g46770* in stamens and carpels was not observed before stage 7 (Figure 5H). Taken together, the results of the in situ hybridization experiments lend further credence to the validity of our approach and demonstrate the usefulness of the 35S:AP1-GR *ap1 cal* system for the identification of genes with distinct expression patterns during early flower development.

### Gene Expression Dynamics

Among the differentially expressed genes identified in the experiment we found an approximately equal number of genes that were activated or repressed. However, we detected considerable differences in the gene expression dynamics between different developmental stages. During the first day after AP1-GR activation, the expression of a large number of genes was down-regulated whereas comparatively few genes were activated (Figure 3B), indicating that the onset of flower development is accompanied by the repression of many genes. This ratio subsequently shifted and from day 3 to day 5, considerably more genes were activated than repressed (Figure 3B). This shift coincided with the initiation of organ primordia and likely marks the activation of specific sets of genes in the developing floral organs [16]. Data from a recent study of xylem vessel element formation also showed a predominant down-regulation of genes at early developmental stages, followed by a predominance of gene activation [34]. Thus, gene repression preceding gene activation upon pathway induction might be a common feature of developmental processes in plants.

The observed predominance of gene repression during the onset of flower formation is in agreement with the findings of a previous study, which identified a large group of genes as repressed in the *Arabidopsis* shoot apex (which is composed of the shoot apical meristem, leaf and floral primordia) upon floral induction after a shift from short day to long day conditions [17]. However, the exact region(s) of the shoot apex in which the identified genes were repressed remained unspecified. Our results strongly suggest that primary sites for gene repression in the shoot apex are those cells of the shoot apical meristem that will give rise to floral primordia, and that the down-regulation of these genes depends, directly or indirectly, on AP1 activity. The limited overlap between the datasets from our study and that of the previous one (Figure S2) implies, however, that gene repression upon floral induction might not be limited to incipient floral primordia but might occur in other parts of the shoot apex as well.

### Transcriptome Analysis

Only a minority of genes that showed differential expression during early flower development were also identified in one of our previous studies as having floral organ-specific expression [16] (Figure S3). Because the vast majority of those organ-specific transcripts are expressed in the reproductive floral organs, and are likely primarily involved in sporogenesis [16] (a process that occurs relatively late in flower development), the limited overlap between the datasets of our two studies is likely a consequence of an extreme specialization of the floral transcriptome during gametophyte formation.

We found a larger (but not an extensive) overlap (Figure S4A) of our dataset with a list of genes that had been previously predicted as being expressed in stamen and carpel primordia formed after a specific activation of the floral homeotic factor AG in *ap1 cal* inflorescences [14]. However, the time points (or developmental stages) at which differential expression was first detected for these genes varied considerably between the two studies. For example, the majority of genes that, in the AG experiment, were detected as differentially expressed 7 d after AG activation (when stamen and carpel development had progressed at least as far as in the oldest floral buds included in this study) showed significant expression changes already within the first 2 d after activation of AP1 (Figure S4B). To rule out the possibility that these discrepancies were due to precocious effects on gene expression caused by AP1-GR activation, we included several of the genes that had been identified in both studies in the in situ hybridization experiments outlined above (Figures 5 and 6), and found that their expression patterns were in good agreement with our microarray results, thus confirming the validity of the information on temporal gene expression that we have obtained.

Clustering of the genes in the dataset based on their expression profiles revealed groups of genes expressed predominantly during distinct stages of early flower development (Figure 3C). We analyzed these groups of co-expressed genes with respect to the distribution of functional categories using Gene Ontology (GO) annotations and found that genes encoding transcription factors were over-represented in all groups. In total, 222 genes, or 13.4% of the genes in the dataset, encode transcription factors (Table S3) compared to ~6% in the *Arabidopsis* genome [35], representing a statistically significant enrichment (*p* value <1 × 10^−4^; χ^2^-test). We found most of the known floral regulators among the transcription factor-coding genes (see above). However, the majority of these genes had not been associated with flower development before, implying that the gene regulatory network underlying early flower development is far more complex than previously thought [14].

We next analyzed the distribution of members of gene families among the transcription factor-coding genes to determine whether certain classes of regulatory genes have been co-opted during evolution to control early flower development. MIKCc-type MADS-box transcription factors are a prominent example of such genes, as many of the floral regulators identified to date encode members of this family [36]. Seventeen (of 39) MIKCc-type MADS-box transcription factors were present in the dataset, representing a statistically significant enrichment (*p* value < 0.001; χ^2^-test). In addition, we found that homeodomain proteins were slightly overrepresented compared to their genome-wide distribution (21 family members compared to 90 genome-wide; *p* value <0.05; χ^2^-test). Furthermore, seven of 11 class II TCP transcription factors were present among the differentially expressed genes (*p* value <0.001; χ^2^-test). While genes encoding C2H2 zinc-finger transcription factors were not overrepresented in the dataset, we found that all but one of the 18 family members identified in the experiment belong to two distinct subgroups (as defined in [37]). Ten genes are members of the C1-1i subgroup, which includes several known floral regulators, namely *JAG, NUB, KNUCKLES (KNU), SUPERMAN (SUP),* and *RABBIT EARS (RBE).* Seven genes belong to the A1a subgroup, none of which has been before associated with flower development.

**Figure 7:**
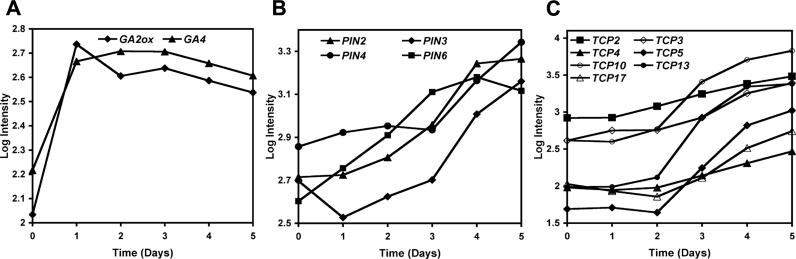
Co-Expression of Genes during Early Flower Development Log_10_-transformed signal intensities at the individual time points are shown for selected genes. (A) Expression profiles for *GA4 (At1g15550)* and a gene *(At1g78440)* encoding a GA2-oxidase. (B) Expression profiles for four genes, encoding members of the PINFORMED (PIN) family of putative auxin efflux carriers. (C) Expression profiles for seven genes, encoding closely related class II TCP-family transcription factors.

We next searched in the dataset for genes involved in defined biological processes and found a considerable number of genes that encode proteins with known roles in the metabolism of, or in the response to, the plant hormones auxin and gibberellin (GA) (Table S4). Both hormones had been previously implicated in mediating distinct processes during flower development [38,39]. For example, the floral homeotic factor AG activates *GA4,* a gene encoding a GA3-β-hydroxylase that catalyzes the formation of biologically active GA, suggesting that AG induces GA biosynthesis during early flower development [14]. In addition to a rapid activation of *GA4,* we found an up-regulation of a gene encoding a GA2-oxidase, which is involved in the degradation of GA and thus counteracts GA4 activity. The expression profiles of both genes were highly correlated (correlation coefficient of 0.96; Figure 7A), suggesting that the regulation of GA levels in floral meristems is a complex process that involves both positive and negative components. Genes involved in auxin production, transport, or response exhibited in general uncorrelated expression profiles. Exceptions were detected for four members of the PINFORMED family of putative auxin efflux carriers (Figure 7B). These proteins are thought to mediate polar auxin transport, a process that is involved in primordial patterning and outgrowth [40]. The concomitant upregulation of these genes at a developmental stage when floral organs arise suggests their possible involvement in the auxin-mediated initiation of floral organ primordia.

### Floral Organ Specification

A key event during early flower development is the specification of the different types of floral organs, a process that is mediated by the floral homeotic genes (see above). These genes encode transcription factors that act in a combinatorial manner to control the developmental programs required for organ formation [41–44]. In spite of extensive efforts [14,15,45,46], only few target genes of these factors have been identified to date (Figure 1), so that the developmental mechanisms by which these factors control organogenesis have remained largely elusive. The known direct target genes of the floral homeotic factors have in common that their promoters contain the previously identified binding site (the so-called CArG box; consensus: 5′-CC(A/T)_6_GG-3′) for members of the MADS box transcription factor family [14,15,47,48], to which most of the floral homeotic factors belong.

As the floral homeotic genes were rapidly activated after the synchronized induction of flower development (Figure 4B), we expected to find their known target genes among the genes that showed significant expression changes in our experiment. Indeed, most of these genes were present in our dataset (Table S5). This result suggests that additional target genes of the floral homeotic factors are likely among the genes we identified as differentially expressed in our experiment. In an attempt to identify candidates for such genes, we searched in the promoters of the differentially expressed genes for the occurrence of CArG box-like sequences (Table S6). Because of the predominant role that the floral homeotic factors play during early flower development, we expected to find these sites to be over-represented in the dataset. However, the occurrence of CArG boxes in the promoters of the genes we identified in the experiment was not significantly different compared to their genome-wide distribution (Table S6). Thus, the floral homeotic factors might either have a limited number of target genes during early flower development, or they might be able to bind to sites other, or less conserved, than the CArG box consensus sequence we have screened for in our analysis.

### Enrichment of Members of Gene Families in Groups of Co-Expressed Genes

Because members of gene families have been reported to be frequently expressed in the same tissues [11], we searched for genes in the dataset that might have been co-opted for specific developmental processes during early flower development. To this end, we analyzed the occurrence of closely related sequences in the dataset (see Materials and Methods for details). We focused initially on genes encoding transcription factors and identified 37 groups that contained two or more closely related sequences. Overall, these groups comprised 91 of the 222 identified transcription factors (Table S7), representing a significant enrichment compared to the mean number of closely related sequences in (equally sized) groups of transcription factors randomly selected from the *Arabidopsis* transcriptome (54.1 ± 8.5 s.d.). We next determined whether the related genes we identified had similar expression profiles. To this end, we determined pair-wise correlation coefficients for the genes in each group and found that almost half (40 of 87 possible comparisons) of the gene pairs had highly correlated expression profiles (Figure S5). Among these genes, a group of seven closely related members of the plant-specific family of TCP transcription factors stood out because all possible pair-wise comparisons resulted in significant correlation values (Figure 7C). For two of these factors, TCP2 and TCP3, similar expression patterns in developing floral organs had been previously reported [49]. Notably, four of the seven co-expressed TCP transcription factors are regulated by microRNAs (miRNAs) [50]. Overexpression of a miRNA that targets these genes led to a strong reduction of their mRNA levels resulting in a severe leaf phenotype but not in a marked disruption of flower development [50]. Our results suggest that this lack of a floral phenotype might be caused by a high degree of functional redundancy among the co-expressed TCP transcription factors, several of which are not subjected to miRNA-mediated regulation.

The family of TCP transcription factors is subdivided into two classes. According to a recent model for TCP transcription factor function, class I TCP factors promote cell growth and division in young organ primordia while members of class II negatively regulate cell proliferation at more advanced developmental stages when organs primarily grow through cell elongation [51]. In agreement with this model, we found that all of the co-expressed TCP factors, whose expression gradually increased during the course of the experiment, belong to class II and thus might contribute to a reduction of cell proliferation rates in maturing floral organs.

**Figure 8:**
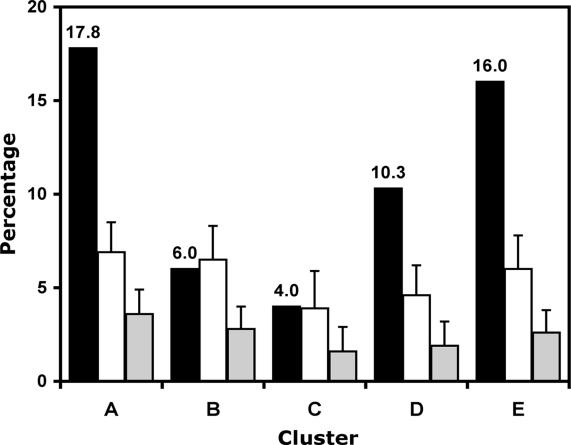
Occurrence of Related Sequences in Groups of Co-Expressed Genes The proportion of closely related sequences in each of the five clusters shown in Figure 3C is indicated by black bars. White and gray bars represent the proportion of closely related sequences in (equally sized) sets of sequences randomly chosen from the list of 1,653 differentially expressed genes and the *Arabidopsis* genome, respectively. Bars indicate the standard deviation of the calculations (see Materials and Methods for details). Note the strong enrichment of related sequences in clusters A, D, and E.

We next broadened the scope of our analysis of closely related sequences in the groups of co-expressed genes, to include not only transcription factor-coding genes but rather all genes regardless of their functional classification. We found an enrichment of closely related members of gene families in all co-expression groups compared to random sets of genes (Figure 8 and Table S8). In particular, the proportion of sequence-related genes in three of these groups was significantly (i.e. beyond three standard deviations) above the already elevated background distribution of related sequences in the dataset (Figure 8). One of these groups comprised genes that are strongly expressed in *ap1 cal* meristems and whose expression is rapidly down-regulated upon AP1-GR activation (cluster A in Figure 3C). The other two groups contained genes that are strongly expressed at stages 4–7 and 5–7 (clusters D and E in Figure 3C), respectively, and are likely predominantly expressed in the developing floral organs (see above). In contrast, genes with high expression during the earliest floral stages (clusters B and C in Figure 3C) showed no significant enrichment of related sequences compared to their background distribution in the dataset. Because members of gene families in *Arabidopsis* frequently act in a redundant manner [7], these stage-specific differences in the occurrence of closely related sequences suggest varying degrees of functional redundancy during early flower development. A high degree of functional redundancy in shoot meristems and developing floral organs might also, at least partially, account for the limited number of mutants identified to date that exhibit specific defects in the development of these structures.

Functional redundancy is thought to serve as a genetic buffering mechanism to increase the robustness of biological systems [52]. Shoot apical meristems are prime examples of robust systems because they maintain their function and size throughout plant development [53]. A high degree of functional redundancy in shoot apical meristems might therefore be a mechanism that protects these essential structures, from which all above ground organs of plants are derived, from disruptive effects of mutations. The same idea might apply to genes activated during floral organ development. Analysis of genes with specific expression in the different types of floral organs showed that the vast majority of these genes is expressed in stamens and carpels, in agreement with the highly complex architecture of these organs compared to that of sepals and petals [16]. Because normal development of the reproductive floral organs is essential for plant propagation, genetic buffering by functional redundancy might be highly beneficial, so that duplicated genes are retained by positive selection [6].

Our analysis provides a detailed description of the gene expression dynamics during early flower development and complements other efforts to study gene expression during *Arabidopsis* development on a genome-wide scale. This information should allow a systematic reverse genetic approach to identify novel regulators that control different aspects of flower development. Because our results suggest that functional redundancy among closely related genes plays a major role in flower development, the simultaneous inactivation of several related genes will likely be necessary to uncover the function of many of the identified genes in flower formation. The floral induction system we have described should facilitate the dissection of the gene regulatory network underlying flower development by allowing the collection of sufficient plant material for approaches such as the genome-wide localization of transcription factor binding sites by chromatin immunoprecipitation.

## Materials and Methods

### Strains and plant growth.

The 35S:AP1-GR *ap1 cal* line was constructed by crossing a previously described 35S:AP1-GR transgene [22] into the *ap1–1 cal-1* double mutant background [19]. For in situ hybridizations, wild-type plants of the accession Landsberg *erecta* were used. Plants were grown on a soil:vermiculite:perlite mixture under constant illumination at 20 °C.

### Microarray setup.

Microarrays were based on the *Arabidopsis* Genome Oligo Set Version 1.0 and on the *Arabidopsis* Genome Oligo Set Version 1.0 Upgrade (Operon, Alameda, California, United States). These sets consist of a total of 30,194 oligonucleotides that correspond to 26,753 annotated genes. Microarrays were manufactured as previously described [16].

### Tissue collection and microarray experiments.

Immediately after the onset of bolting, inflorescences of 35S:AP1-GR *ap1 cal* plants were treated with a solution containing 1 μM dexamethasone (Sigma-Aldrich, St. Louis, Missouri, United States), 0.01% (v/v) ethanol, and 0.015% (v/v) Silwet L-77. For each time point, tissue from ~25 plants was collected using jewelers forceps. Tissue was removed as close to the surface of the inflorescence as possible to ensure an enrichment of meristematic cells.

The methods used for extracting total RNA from tissue samples, for the amplification of mRNA, and for the labeling of RNA samples with fluorescent dyes have been previously described [16]. Hybridizations were done as follows: dye-labeled antisense RNA preparations were dried down and the resulting pellets were re-suspended in 5 μl 10 mM EDTA and 45 μl SlideHyb Buffer #1 (Ambion, Austin, Texas, United States) and hybridized for 14 h to microarrays at 48 °C using a MAUI hybridization system (BioMicro Systems, Salt Lake City, Utah, United States) according to the manufacturer's instructions.

Four independent sets of biological samples were used for the experiments. The samples derived from consecutive time points were co-hybridized, resulting in a total of five hybridizations per set. The dyes used for labeling RNA from a given time point were switched in the replicate experiments to reduce dye-related artifacts.

### Data analysis.

Microarrays were scanned with a GenePix 4200A scanner (Axon Instruments, Foster City, California, United States) as previously described [16] using the Gene Pix 5.0 analysis software (Axon Instruments). Raw data were imported into the Resolver gene expression data analysis system version 4.0 (Rosetta Biosoftware, Seattle, Washington, United States) and processed as described [16]. Because the statistical model used by Resolver v4.0 does not account for multihypothesis testing, we adjusted the *p* values calculated by this software for each time point using the Holm procedure as implemented in the Bioconductor *multtest* package (http://www.bioconductor.org/packages/bioc/stable/src/contrib/html/multtest.html). The Holm procedure allows a strong control over family-wise type I errors (“false positives”). Genes for which the adjusted *p* value was <0.05 in at least one of the comparisons were considered differentially expressed in the experiment. No fold-change cut-off was applied. Intensity values were derived from the ratio data for each of the hybridizations, using the Ratio-Split function of Resolver v5.0. The intensity data of the replicate experiments were subsequently combined, resulting in six datasets corresponding to the individual time points of the experiment. All analyses in Resolver were done at the so-called sequence level, i.e., data from reporters (probes) representing the same gene were combined.

Microarray data from the experiments by Schmid et al. [17] were obtained as .cel files and processed in Resolver v4.0. In order to identify genes with significant expression changes, Analysis of Variance (ANOVA) with error weighting (using estimated platform-specific measurement errors) was performed for each of the experiments. ANOVA *p* values were adjusted for multihypothesis testing as outlined above. Genes for which the adjusted ANOVA *p* value was <0.05 were considered as differentially expressed.

Pair-wise Pearson correlation coefficients (ρ) were calculated using log_2_-transformed signal intensities. Correlation coefficients with an absolute value of >0.811 were considered statistically significant assuming an alpha level of 0.05 and four degrees of freedom.

Groups of co-expressed genes were identified using the k-means algorithm implemented in Resolver with z-score normalized signal intensities for the differentially expressed genes as input values.

The identification of closely related sequences in the dataset was based on BLASTP [54]. In a first step, pairs of related sequences in an analysis group were identified. To this end, each sequence was compared to all available *Arabidopsis* protein sequences and the resulting hits were searched for the occurrence of another member of the analysis group using a rank cut-off of 5 (excluding the best hit, which was identical to the input sequence) and an e-value cut-off of 1 × 10^−20^. Sequence pairs were further combined into clusters with more than two (non-redundant) sequences if the sequences of that cluster resulted in reciprocal hits. For the calculation of background distributions of closely related sequences, 100 random sets of sequences were generated containing the same number of sequences as the corresponding analysis group. These sets were analyzed using BLASTP as outlined above. The mean number of related sequences as well as the standard deviation were calculated for each group using the results from the individual BLASTP searches. Sequences used for BLASTP were obtained from The *Arabidopsis* Information Resource (TAIR) (http://www.arabidopsis.org).

Each probe represented on the microarrays used for our experiments was designed to specifically detect the transcript of a single gene. However, in some cases, when the sequence identity of genes is very high, gene-specific probes cannot be designed and cross-hybridization between related mRNA species might occur. To test whether the identification of closely related sequences in groups of co-expressed genes had been significantly affected by non-gene-specific probes, we analyzed the potential for cross-hybridization for those probes representing the genes described in Table S8. To this end, we considered probes with 70% or more sequence identity to genes other than their intend target as potentially non-specific [55]. We found only a small number of genes (five out of 196) whose microarray results might have been affected by cross-hybridization (unpublished data). Thus, we concluded that our analysis was not significantly influenced by non-specific probes.

For the identification of functionally related genes and of genes involved in the same biological process, we obtained GO predictions from TAIR and then searched for statistically overrepresented GO terms using the program GOToolBox [56] (http://crfb.univ-mrs.fr/GOToolBox/index.php). We also used gene family information and gene annotations from TAIR.

### Promoter analysis.

For the identification of CArG box sequences in the promoters of the differential expressed genes, we used the program Patmatch (http://www.arabidopsis.org/cgi-bin/patmatch/nph-patmatch.pl). We searched the 500-bp and 1,000-bp regions preceding the 5′ end of a transcription unit (TAIR datasets “Loci Upstream Sequences-500bp” and “Loci Upstream Sequences-1000bp”, respectively), as well as the 1000-bp region downstream of a transcription unit (TAIR dataset “Loci Downstream Sequences-1000bp”) for the occurrence of the CArG box consensus (5′-CC(A/T)_6_GG-3′). We also screened the 500-bp upstream regions for CArG box-like sequences, allowing one nucleotide substitution compared to the CArG box consensus.

### In situ hybridization.

Non-radioactive in situ hybridizations were performed as previously described [57] (a detailed in situ protocol can be found at http://www.its.caltech.edu/~plantlab/html/protocols.html). Primers used for the amplification of cDNA fragments for the genes tested are listed in Table S9. PCR products were ligated into pGEM-T Easy (Promega, Madison, Wisconsin, United States) by TA cloning, and the resulting vectors were sequenced to determine the orientation of the inserts.

### Scanning electron microscopy.

Inflorescences from 35S:AP1-GR *ap1 cal* plants were collected at different time points after dexamethasone treatment. The samples were processed for scanning electron microscopy as previously described [43].

## Supporting Information

Figure S1 Activation of AP1-GR in Wild-Type Plants Activation of AP1-GR in wild-type plants causes the transformation of inflorescence (A) or vegetative shoot meristems (B) into floral meristems, leading to the formation of terminal flowers. (A) Inflorescence was treated daily for 1 wk with a solution containing 10 μM dexamethasone. Image was taken 14 d after the first treatment. (B) Plant was germinated on a plate with medium containing 50 nM dexamethasone. Image was taken 21 d post germination.
(987 KB PDF)

Figure S2 Overlap between Genes Detected as Differentially Expressed upon AP1-GR Activation and upon Floral Induction Genes detected as differentially expressed upon AP1-GR activation were compared to genes identified by Schmid et al. [17] as significantly changed in *Arabidopsis* shoot apices after floral induction. Genes identified in the latter study belong to at least three classes: genes expressed in leaf primordia; genes expressed in floral primordia; and genes whose expression changes in shoot meristems after floral induction. (A) A Venn diagram depicts the overlap between the differentially expressed genes identified upon AP1-GR activation (r_1_-r_5_) and the ‘Top 500 list' described in Schmid et al. [17], which represents the overlap between the 500 most significantly changed genes in wild-type plants of the accessions Landsberg *erecta* (L-er) and Columbia (Col), respectively. (B) Data by Schmid et al. [17] were reanalyzed as outlined in Materials and Methods to allow a detailed comparison of the experimental results. The overlap between the different datasets is shown. Numbers in parenthesis indicate the total number of genes in each dataset.
(54 KB PDF)

Figure S3 Overlap between Genes Detected as Differentially Expressed upon AP1-GR Activation and Genes Expressed in the Different Types of Floral Organs Genes detected as differentially expressed upon AP1-GR activation (r_1_-r_5_) were compared to genes identified as being specifically or predominantly expressed in sepals, petals, stamens, or carpels [16]. Numbers in parenthesis indicate the total number of genes in each group.
(37 KB PDF)

Figure S4 Comparison of AP1-GR and AG-GR Datasets Comparison between genes detected as differentially expressed upon AP1-GR activation and genes identified by Gomez-Mena et al. [14] as significantly changed in *ap1 cal* inflorescences upon activation of an AG-GR fusion protein, which leads to the formation of stamens and carpels. (A) A Venn diagram depicts the overlap between the differentially expressed genes identified upon AP1-GR activation (r_1_-r_5_) and the genes described by Gomez-Mena et al. (AG-GR). Numbers in parenthesis indicate the total number of genes in each dataset. (B) Temporal distribution of gene expression changes. Genes that were identified in both studies were analyzed with respect to the time point at which differential expression was first detected.
(59 KB PDF)

Figure S5 Examples for Closely Related Transcription Factors with Similar Expression Profiles during Early Flower Development (A) Co-expression of *At2g18550* and *At5g66700,* encoding homeobox-leucine zipper proteins. (B) Co-expression of *FD (At4g35900),* encoding a basic leucine zipper containing factor, and its paralog *FDP (At2g17770).* (C) Negative correlation of expression of two genes *(At1g55110* and *At5g03150),* encoding C2H2 zinc-finger domain containing proteins. (D) Co-expression of *ASYMMETRIC LEAVES2-LIKE20/LOB DOMAIN PROTEIN18 (ASL20/LBD18; At2g45420)* and *ASYMMETRIC LEAVES2-LIKE23/ LOB DOMAIN PROTEIN19 (ASL23/LBD19; At2g45410). ASL20* and *ASL23* are arranged in tandem. The correlation coefficient ρ is indicated for each pairwise comparison. Log_10_-transformed signal intensities at the individual time points of the experiment are shown.
(171 KB PDF)

Table S1 Known Floral Regulatory Genes Identified as Differentially Expressed in the Experiment Gene identifiers and gene names are listed. Literature describing the expression of the genes during early flower development is referenced.
(51 KB PDF)

Table S2 Differentially Expressed Genes Inferred from Ratios r_1_-r_5_ (see Figure 3A) Gene identifiers and gene descriptions are shown. Gene descriptions were derived from various sources, including information from TAIR. The assignment of the individual genes to the clusters (A–E) of co-expressed genes shown in Figure 3C is indicated in the column “Cluster”. Fold change values and adjusted *p* values (“Holm”) for ratios r_1_-r_5_, as well as normalized signal intensities for the different time points (0–5 d) are shown.
(848 KB XLS)

Table S3 Transcription Factors Identified in the Dataset Gene identifiers, gene names, and gene descriptions are shown. Gene family information is based on [35]. The assignment of the individual genes to the clusters (A–E) of co-expressed genes shown in Figure 3C is indicated in the column “Cluster”.
(63 KB XLS)

Table S4 Differentially Expressed Genes That Are Involved in the Metabolism of, or in the Response to, the Plant Hormones Gibberellin, and Auxin, Respectively Gene identifiers and gene descriptions are listed.
(20 KB XLS)

Table S5 Known or Presumed Target Genes of Floral Homeotic Factors Detected as Differentially Expressed in the Experiment Gene identifiers and gene names (or descriptions) are listed, and relevant literature is referenced.
(43 KB XLS)

Table S6 Distribution of CArG Box Sequences in the Promoters of Genes Identified as Differentially Expressed during Early Flower Development Four different analyses were performed as outlined in Materials and Methods. The spreadsheet “CArG” lists the number of sites found in each of the analyzed promoter regions. Gene identifiers and gene descriptions of the corresponding genes are shown. The assignment of the individual genes to the clusters (A–E) of co-expressed genes shown in Figure 3C is indicated in the column “Cluster”.
The spreadsheet “Binding Sites” summarizes the results of the analysis. On the left, the frequency of CArG boxes in the dataset is compared to their genome-wide distribution. On the right, the number of genes with CArG boxes in each of the groups of co-expressed genes shown in Figure 3C is listed. Numbers in parenthesis indicate the expected number of genes with CArG boxes based on their genome-wide distribution.
(462 KB XLS)

Table S7 Groups of Closely Related Transcription Factors in the Dataset Gene identifiers, gene names, and gene descriptions are shown. Gene family information is based on [35].
(34 KB XLS)

Table S8 Groups of Closely Related Members of Gene Families Identified in the Clusters of Co-Expressed Genes Shown in Figure 3C Gene identifiers and gene descriptions are listed.
(64 KB XLS)

Table S9 Primers Used to Generate Probes for In Situ Hybridizations Gene identifiers and corresponding primer sequences are shown.
(33 KB XLS)

Table S10 Experimental Data for All Genes Represented on the Microarrays Used in this Study Gene identifiers and gene descriptions are shown. Gene descriptions were derived from various sources, including information from TAIR. Fold change values and adjusted *p* values ('Holm') for ratios r_1_-r_5_ (see Figure 3A), as well as normalized signal intensities for the different time points (0–5 d) are shown.
(12 MB XLS)

Table S11 Selected References for the Gene Interactions Summarized in the Network Diagram Shown in Figure 1
The mode of an interaction (direct or indirect) is specified, if known. Abbreviations: AG: AGAMOUS; AGL24: AGAMOUS-LIKE24; ANT: AINTEGUMENTA; AP1: APETALA1; AP2: APETALA2; AP3: APETALA3; ASK1: ARABIDOPSIS SKP-LIKE1; BLR: BELLRINGER; CAL: CAULIFLOWER; CLF: CURLY LEAF; CLV3: CLAVATA3; CRC: CRABS CLAW; FT: FLOWERING LOCUS T; FUL: FRUITFULL; GA4: GA REQUIRING 4; LEU: LEUNIG; LFY: LEAFY; miR172: microRNA172; NAP: NAC-LIKE, ACTIVATED BY AP3/PI; NZZ/SPL: NOZZLE/SPOROCYTELESS; PI: PISTILLATA; RBE: RABBIT EARS; SAP: STERILE APETALA; SEP1–4: SEPALLATA1–4; SEU: SEUSS; SHP2: SHATTERPROOF2; SUP:SUPERMAN; TFL1: TERMINAL FLOWER 1; UFO: UNUSUAL FLORAL ORGANS; WUS: WUSCHEL.
(41 KB PDF)

### Accession Numbers

Microarray data have been deposited with the NCBI Gene Expression Omnibus (http://www.ncbi.nlm.nih.gov/geo) under accession number GSE4594. Ratio and intensity data for all genes represented on the microarray used in this study are listed in Table S10.

The Arabidopsis Genome Initiative (http://www.arabidopsis.org) identifiers for the genes and gene products discussed in this study are: AG (At4g18960), AGL24 (At4g24540), AGL42 (At5g62165), ANT (At4g37750), AP1 (At1g69120), AP2 (At4g36920), AP3 (At3g54340), ASK1 (At1g10940), BLR (At5g02030), CAL (At1g26310), CLF (At2g23380), CLV3 (At2g27250), CRC (At1g69180), FD (At4g35900), FDP (At2g17770), FIL (At2g45190), FT (At1g65480), FUL (At5g60910), GA2ox1 (At1g78440), GA4 (At1g15550), JAG (At1g68480), KNU (At5g14010), LEU (At4g32550), LFY (At5g61850), NAP (At1g69490), NUB (At1g13400), NZZ/SPL (At4g27330), PI (At5g20240), PIN2 (At5g57090), PIN3 (At1g70940), PIN4 (At2g01420), PIN6 (At1g77110), RBE (At5g06070), SAP (At5g35770), SEP1 (At5g15800), SEP2 (At3g02310), SEP3 (At1g24260), SEP4 (At2g03710), SEU (At1g43850), SHP1 (At3g58780), SHP2 (At2g42830), SOC1 (At2g45660), SUP (At3g23130), SVP (At2g22540), TCP2 (At4g18390), TCP3 (At1g53230), TCP4 (At3g15030), TCP5 (At5g60970), TCP10 (At2g31070), TCP13 (At3g02150), TCP17 (At5g08070), TFL1 (At5g03840), UFO (At1g30950), WUS (At2g17950), and YAB3 (At4g00180).

## Abbreviations

*AG*: *AGAMOUS*
*AGL24*: *AGAMOUS-LIKE 24*
*AGL42*: *AGAMOUS-LIKE 42*
ANOVA: Analysis of Variance
*AP1*: *APETALA1*
*AP2*: *APETALA2*
*AP3*: *APETALA3*
*CAL*: *CAULIFLOWER*
*CRC*: *CRABS CLAW*
*CLV3*: *CLAVATA3*
*FIL*: *FILAMENTOUS FLOWER*
*FUL*: *FRUITFULL*
GA: gibberellic acid
GR: glucocorticoid receptor
GO: Gene Ontology
*JAG*: *JAGGED*
*KNU*: *KNUCKLES*
*LFY*: *LEAFY*
miRNA: microRNA
*NZZ*: *NOZZLE*
*NUB*: *NUBBIN*
*PI*: *PISTILLATA*
*RBE*: *RABBIT EARS*
*SHP1/2*: *SHATTERPROOF 1* and *2;*
*SPL*: *SPOROCYTELESS*
*SOC1*: *SUPPRESSOR OF CO-OVEREXPRESSION 1*
*SVP*: *SHORT VEGETATIVE PHASE*
*SUP*: *SUPERMAN*
TAIR: The *Arabidopsis* Information Resource
*TFL1*: *TERMINAL FLOWER 1*
*WUS*: *WUSCHEL*
*YAB3*: *YABBY3*
