# Supplementary figures and images for: Genome-Wide Analysis of Gene Expression during Early Arabidopsis Flower Development
Source: PLoS Genet. 2006 Jul 28;2(7):e117. doi: 10.1371/journal.pgen.0020117 (PMC1523247; doi:10.1371/journal.pgen.0020117)

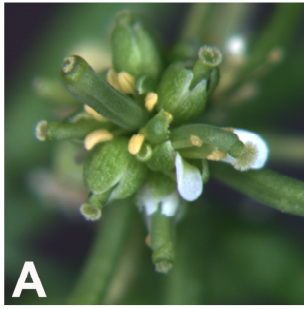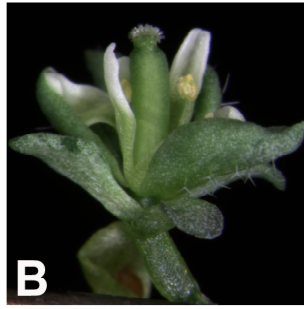

Supplement: Figure S1 — Activation of AP1-GR in wild-type plants causes the transformation of inflorescence (A) or vegetative shoot meristems (B) into floral meristems, leading to the formation of terminal flowers. (A) Inflorescence was treated daily for 1 wk with a solution containing 10 μM dexamethasone. Image was taken 14 d after the first treatment. (B) Plant was germinated on a plate with medium containing 50 nM dexamethasone. Image was taken 21 d post germination. (987 KB PDF) [file pgen.0020117.sg001.pdf]

**A**

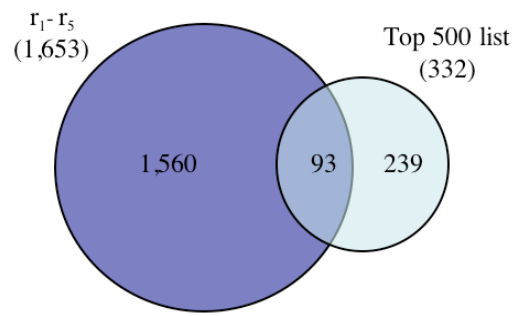

**B**

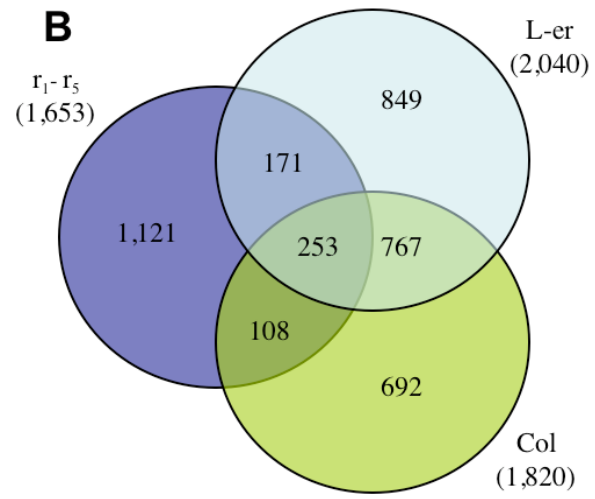

Supplement: Figure S2 — Genes detected as differentially expressed upon AP1-GR activation were compared to genes identified by Schmid et al. [17] as significantly changed in Arabidopsis shoot apices after floral induction. Genes identified in the latter study belong to at least three classes: genes expressed in leaf primordia; genes expressed in floral primordia; and genes whose expression changes in shoot meristems after floral induction. (A) A Venn diagram depicts the overlap between the differentially expressed genes identified upon AP1-GR activation (r1-r5) and the ‘Top 500 list' described in Schmid et al. [17], which represents the overlap between the 500 most significantly changed genes in wild-type plants of the accessions Landsberg erecta (L-er) and Columbia (Col), respectively. (B) Data by Schmid et al. [17] were reanalyzed as outlined in Materials and Methods to allow a detailed comparison of the experimental results. The overlap between the different datasets is shown. Numbers in parenthesis indicate the total number of genes in each dataset. (54 KB PDF) [file pgen.0020117.sg002.pdf]

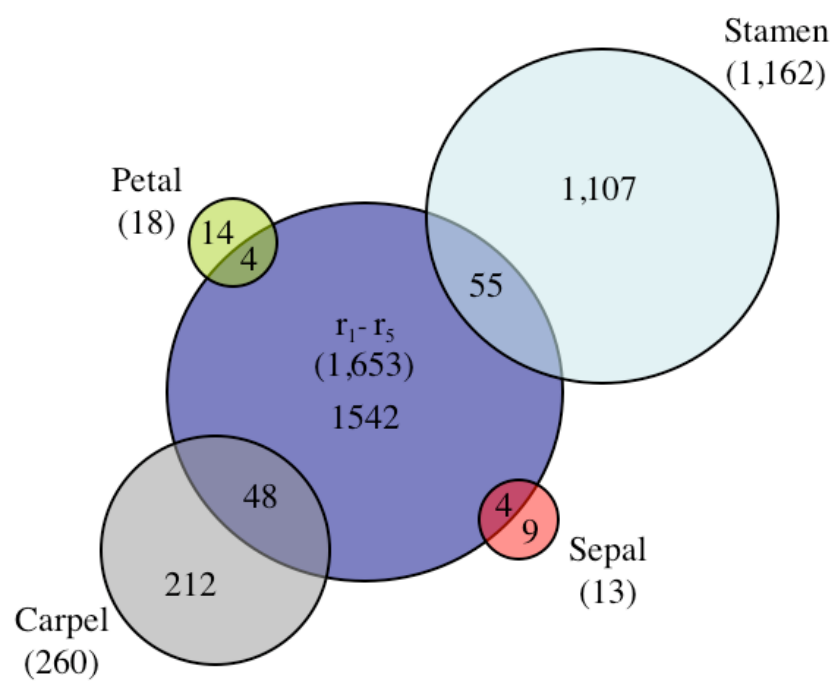

Supplement: Figure S3 — Genes detected as differentially expressed upon AP1-GR activation (r1-r5) were compared to genes identified as being specifically or predominantly expressed in sepals, petals, stamens, or carpels [16]. Numbers in parenthesis indicate the total number of genes in each group. (37 KB PDF) [file pgen.0020117.sg003.pdf]

**A**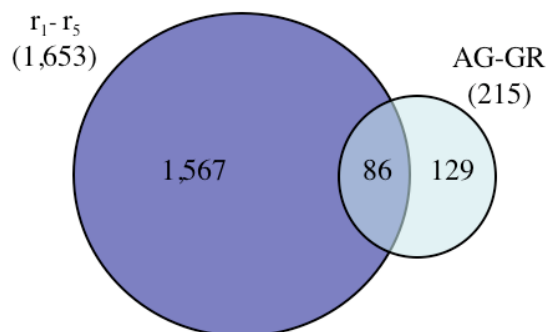**B**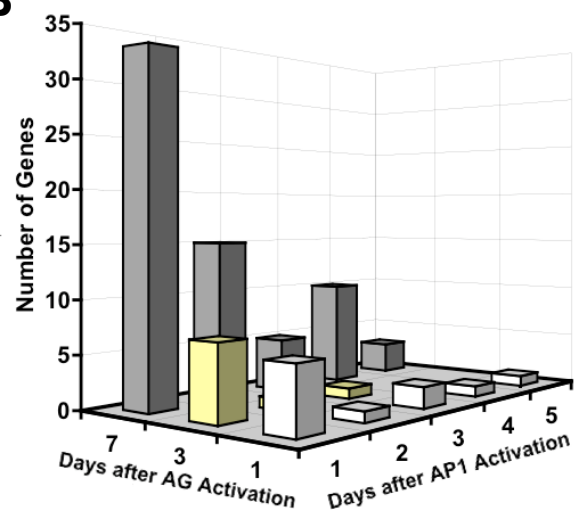

Supplement: Figure S4 — Comparison between genes detected as differentially expressed upon AP1-GR activation and genes identified by Gomez-Mena et al. [14] as significantly changed in ap1 cal inflorescences upon activation of an AG-GR fusion protein, which leads to the formation of stamens and carpels. (A) A Venn diagram depicts the overlap between the differentially expressed genes identified upon AP1-GR activation (r1-r5) and the genes described by Gomez-Mena et al. (AG-GR). Numbers in parenthesis indicate the total number of genes in each dataset. (B) Temporal distribution of gene expression changes. Genes that were identified in both studies were analyzed with respect to the time point at which differential expression was first detected. (59 KB PDF) [file pgen.0020117.sg004.pdf]

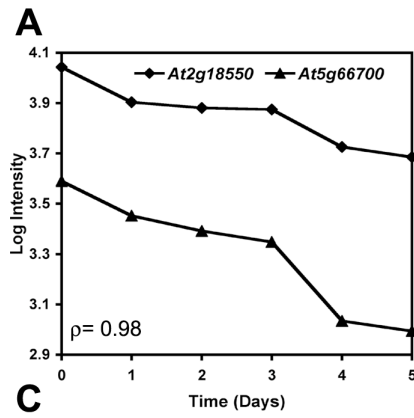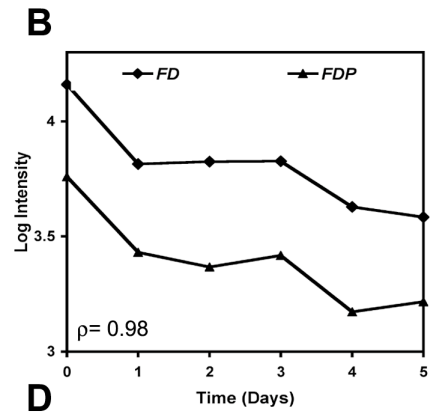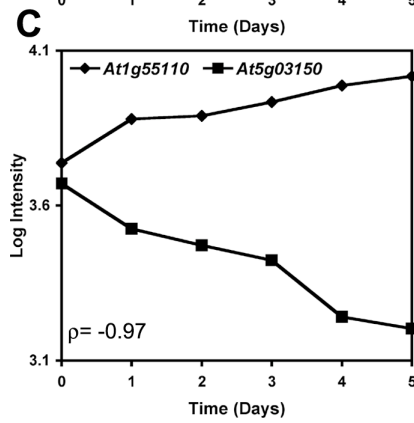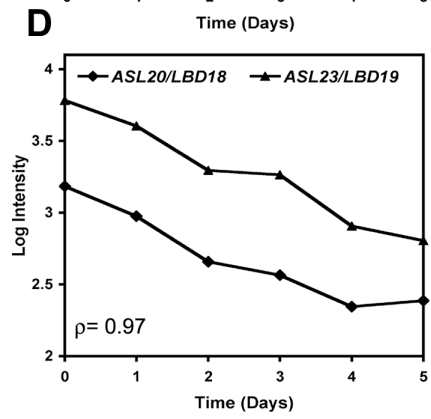

Supplement: Figure S5 — (A) Co-expression of At2g18550 and At5g66700, encoding homeobox-leucine zipper proteins. (B) Co-expression of FD (At4g35900), encoding a basic leucine zipper containing factor, and its paralog FDP (At2g17770). (C) Negative correlation of expression of two genes (At1g55110 and At5g03150), encoding C2H2 zinc-finger domain containing proteins. (D) Co-expression of ASYMMETRIC LEAVES2-LIKE20/LOB DOMAIN PROTEIN18 (ASL20/LBD18; At2g45420) and ASYMMETRIC LEAVES2-LIKE23/ LOB DOMAIN PROTEIN19 (ASL23/LBD19; At2g45410). ASL20 and ASL23 are arranged in tandem. The correlation coefficient ρ is indicated for each pairwise comparison. Log10-transformed signal intensities at the individual time points of the experiment are shown. (171 KB PDF) [file pgen.0020117.sg005.pdf]
