# Supplementary material for: Genome-Wide Analysis of Gene Expression during Early Arabidopsis Flower Development
Source: PLoS Genet. 2006 Jul 28;2(7):e117. doi: 10.1371/journal.pgen.0020117 (PMC1523247; doi:10.1371/journal.pgen.0020117)
Supplement: Table S1 — Gene identifiers and gene names are listed. Literature describing the expression of the genes during early flower development is referenced. (51 KB PDF) [file pgen.0020117.st001.pdf]

Table S1: Known floral regulatory genes identified as differentially expressed in the experiment. Gene identifiers and gene names are listed. Literature describing the expression of the genes during flower development is referenced.

Gene Identifier	Name	Reference
At4g18960	*AGAMOUS*	[1,2]
At4g24540	*AGAMOUS-LIKE 24*	[3]
At4g36920	*APETALA2*	[4]
At3g54340	*APETALA3*	[5]
At1g26310	*CAULIFLOWER*	[6]
At2g27250	*CLAVATA3*	[7]
At1g69180	*CRABS CLAW*	[8,9]
At3g15170	*CUP-SHAPED COTELYDON1*	[10]
At1g76420	*CUP-SHAPED COTELYDON3*	[30]
At1g12980	*ENHANCER OF SHOOT REGENERATION/ DORNROESCHEN*	[11]
At4g35900	*FD*	[13,14]
At2g45190	*FILAMENTOUS FLOWER*	[15]
At5g24860	*FLOWERING PROMOTING FACTOR1*	[16]
At1g68480	*JAGGED*	[17,18]
At5g14010	*KNUCKLES*	[19]
At5g61850	*LEAFY*	[20]
At4g27330	*NOZZLE/SPOROCYTELESS*	[21,22]
At1g13400	*NUBBIN*	[32]
At1g68640	*PERIANTHIA*	[23]
At5g20240	*PISTILLATA*	[24]
At3g59380	*PLURIPETALA*	[41]
At2g28610	*PRESSED FLOWER*	[25]
At5g06070	*RABBIT EARS*	[26]
At1g24260	*SEPALLATA3*	[27]
At2g03710	*SEPALLATA4*	[28]
At2g42830	*SHATTERPROOF2*	[29]
At2g22540	*SHORT VEGETATIVE PHASE*	[31]
At5g35770	*STERILE APETALA*	[12]
At3g23130	*SUPERMAN*	[33,34]
At2g45660	*SUPPRESSOR OF OVEREXPRESSION OF CO 1*	[35]
At5g03840	*TERMINAL FLOWER 1*	[36]
At1g30950	*UNUSUAL FLORAL ORGANS*	[37]
At2g17950	*WUSCHEL*	[38,39,40]
At4g00180	*YABBY3*	[15]

REFERENCES:

1. Ito T, Wellmer F, Yu H, Das P, Ito N, et al. (2004) The homeotic protein AGAMOUS controls microsporogenesis by regulation of SPOROCYTELESS. Nature 430: 356-360.
2. Yanofsky MF, Ma H, Bowman JL, Drews GN, Feldmann KA, et al. (1990) The protein encoded by the Arabidopsis homeotic gene agamous resembles transcription factors. Nature 346: 35-39.
3. Yu H, Ito T, Wellmer F, Meyerowitz EM (2004) Repression of AGAMOUS-LIKE 24 is a crucial step in promoting flower development. Nat Genet 36: 157-161.
4. Jofuku KD, den Boer BG, Van Montagu M, Okamuro JK (1994) Control of Arabidopsis flower and seed development by the homeotic gene APETALA2. Plant Cell 6: 1211-1225.
5. Jack T, Brockman LL, Meyerowitz EM (1992) The homeotic gene APETALA3 of Arabidopsis thaliana encodes a MADS box and is expressed in petals and stamens. Cell 68: 683-697.
6. Kempin SA, Savidge B, Yanofsky MF (1995) Molecular basis of the cauliflower phenotype in Arabidopsis. Science 267: 522-525.
7. Fletcher JC, Brand U, Running MP, Simon R, Meyerowitz EM (1999) Signaling of cell fate decisions by CLAVATA3 in Arabidopsis shoot meristems. Science 283: 1911-1914.
8. Bowman JL, Smyth DR (1999) CRABS CLAW, a gene that regulates carpel and nectary development in Arabidopsis, encodes a novel protein with zinc finger and helix-loop-helix domains. Development 126: 2387-2396.
9. Schmid M, Uhlenhaut NH, Godard F, Demar M, Bressan R, et al. (2003) Dissection of floral induction pathways using global expression analysis. Development 130: 6001-6012.
10. Takada S, Hibara K, Ishida T, Tasaka M (2001) The CUP-SHAPED COTYLEDON1 gene of Arabidopsis regulates shoot apical meristem formation. Development 128: 1127-1135.
11. Kirch T, Simon R, Grunewald M, Werr W (2003) The DORNROSCHEN/ENHANCER OF SHOOT REGENERATION1 gene of Arabidopsis acts in the control of meristem cell fate and lateral organ development. Plant Cell 15: 694-705.
12. Byzova MV, Franken J, Aarts MG, de Almeida-Engler J, Engler G, et al. (1999) Arabidopsis STERILE APETALA, a multifunctional gene regulating inflorescence, flower, and ovule development. Genes Dev 13: 1002-1014.
13. Wigge PA, Kim MC, Jaeger KE, Busch W, Schmid M, et al. (2005) Integration of spatial and temporal information during floral induction in Arabidopsis. Science 309: 1056-1059.
14. Abe M, Kobayashi Y, Yamamoto S, Daimon Y, Yamaguchi A, et al. (2005) FD, a bZIP protein mediating signals from the floral pathway integrator FT at the shoot apex. Science 309: 1052-1056.

15. Siegfried KR, Eshed Y, Baum SF, Otsuga D, Drews GN, et al. (1999) Members of the YABBY gene family specify abaxial cell fate in Arabidopsis. Development 126: 4117-4128.
16. Kania T, Russenberger D, Peng S, Apel K, Melzer S (1997) FPF1 promotes flowering in Arabidopsis. Plant Cell 9: 1327-1338.
17. Dinneny JR, Yadegari R, Fischer RL, Yanofsky MF, Weigel D (2004) The role of JAGGED in shaping lateral organs. Development 131: 1101-1110.
18. Ohno CK, Reddy GV, Heisler MG, Meyerowitz EM (2004) The Arabidopsis JAGGED gene encodes a zinc finger protein that promotes leaf tissue development. Development 131: 1111-1122.
19. Payne T, Johnson SD, Koltunow AM (2004) KNUCKLES (KNU) encodes a C2H2 zinc-finger protein that regulates development of basal pattern elements of the Arabidopsis gynoecium. Development 131: 3737-3749.
20. Weigel D, Alvarez J, Smyth DR, Yanofsky MF, Meyerowitz EM (1992) LEAFY controls floral meristem identity in Arabidopsis. Cell 69: 843-859.
21. Schiefthaler U, Balasubramanian S, Sieber P, Chevalier D, Wisman E, et al. (1999) Molecular analysis of NOZZLE, a gene involved in pattern formation and early sporogenesis during sex organ development in Arabidopsis thaliana. Proc Natl Acad Sci U S A 96: 11664-11669.
22. Yang WC, Ye D, Xu J, Sundaresan V (1999) The SPOROCYTELESS gene of Arabidopsis is required for initiation of sporogenesis and encodes a novel nuclear protein. Genes Dev 13: 2108-2117.
23. Chuang CF, Running MP, Williams RW, Meyerowitz EM (1999) The PERIANTHIA gene encodes a bZIP protein involved in the determination of floral organ number in Arabidopsis thaliana. Genes Dev 13: 334-344.
24. Goto K, Meyerowitz EM (1994) Function and regulation of the Arabidopsis floral homeotic gene PISTILLATA. Genes Dev 8: 1548-1560.
25. Matsumoto N, Okada K (2001) A homeobox gene, PRESSED FLOWER, regulates lateral axis-dependent development of Arabidopsis flowers. Genes Dev 15: 3355-3364.
26. Takeda S, Matsumoto N, Okada K (2004) RABBIT EARS, encoding a SUPERMAN-like zinc finger protein, regulates petal development in Arabidopsis thaliana. Development 131: 425-434.
27. Mandel MA, Yanofsky M (1998) The *Arabidopsis AGL9* MADS-box gene is expressed in young flower promordia. Sex Plant Reprod 11: 22-28.
28. Ditta G, Pinyopich A, Robles P, Pelaz S, Yanofsky MF (2004) The SEP4 gene of Arabidopsis thaliana functions in floral organ and meristem identity. Curr Biol 14: 1935-1940.
29. Savidge B, Rounsley SD, Yanofsky MF (1995) Temporal relationship between the transcription of two Arabidopsis MADS box genes and the floral organ identity genes. Plant Cell 7: 721-733.
30. Vroemen CW, Mordhorst AP, Albrecht C, Kwaaitaal MA, de Vries SC (2003) The CUP-SHAPED COTYLEDON3 gene is required for boundary and shoot meristem formation in Arabidopsis. Plant Cell 15: 1563-1577.

31. Hartmann U, Hohmann S, Nettesheim K, Wisman E, Saedler H, et al. (2000) Molecular cloning of SVP: a negative regulator of the floral transition in Arabidopsis. Plant J 21: 351-360.
32. Dinneny JR, Weigel D, Yanofsky MF (2006) NUBBIN and JAGGED define stamen and carpel shape in Arabidopsis. Development.
33. Ito T, Sakai H, Meyerowitz EM (2003) Whorl-specific expression of the SUPERMAN gene of Arabidopsis is mediated by cis elements in the transcribed region. Curr Biol 13: 1524-1530.
34. Sakai H, Medrano LJ, Meyerowitz EM (1995) Role of SUPERMAN in maintaining Arabidopsis floral whorl boundaries. Nature 378: 199-203.
35. Borner R, Kampmann G, Chandler J, Gleissner R, Wisman E, et al. (2000) A MADS domain gene involved in the transition to flowering in Arabidopsis. Plant J 24: 591-599.
36. Bradley D, Ratcliffe O, Vincent C, Carpenter R, Coen E (1997) Inflorescence commitment and architecture in Arabidopsis. Science 275: 80-83.
37. Lee I, Wolfe DS, Nilsson O, Weigel D (1997) A LEAFY co-regulator encoded by UNUSUAL FLORAL ORGANS. Curr Biol 7: 95-104.
38. Alvarez J, Smyth DR (1999) CRABS CLAW and SPATULA, two Arabidopsis genes that control carpel development in parallel with AGAMOUS. Development 126: 2377-2386.
39. Gross-Hardt R, Lenhard M, Laux T (2002) WUSCHEL signaling functions in interregional communication during Arabidopsis ovule development. Genes Dev 16: 1129-1138.
40. Wellmer F, Riechmann JL, Alves-Ferreira M, Meyerowitz EM (2004) Genome-wide analysis of spatial gene expression in Arabidopsis flowers. Plant Cell 16: 1314-1326.
41. Running MP, Lavy M, Sternberg H, Galichet A, Gruissem W, et al. (2004) Enlarged meristems and delayed growth in plp mutants result from lack of CaaX prenyltransferases. Proc Natl Acad Sci U S A 101: 7815-7820.
