# Supplementary material for: Genome-Wide Analysis of Gene Expression during Early Arabidopsis Flower Development
Source: PLoS Genet. 2006 Jul 28;2(7):e117. doi: 10.1371/journal.pgen.0020117 (PMC1523247; doi:10.1371/journal.pgen.0020117)
Supplement: Table S5 — Gene identifiers and gene names (or descriptions) are listed, and relevant literature is referenced. (43 KB XLS) [file pgen.0020117.st005.pdf]

Table S5: Known or presumed target genes of floral homeotic factors detected as differentially expressed in the experiment. Gene identifiers and gene names (or descriptions) are listed, and relevant literature is referenced.

Gene ID	Name/Description	Reference
At4g18960	*AGAMOUS*	[1]
At3g54340	*APETALA3*	[1,2]
At1g69180	*CRABS CLAW*	[1]
At4g27330	*NOZZLE/SPOROCYTELESS*	[3]
At1g24260	*SEPALLATA3*	[1]
At2g42830	*SHATTERPROOF2*	[4]
At4g32980	*ATH1*	[1]
At3g11000	*Expressed protein*	[1]
At3g17010	*B3 domain containing protein*	[1]
At1g47610	*WD40 domain containing protein*	[1]
At1g13400	*NUBBIN*	[1]

REFERENCES:

1. Gomez-Mena C, de Folter S, Costa MM, Angenent GC, Sablowski R (2005) Transcriptional program controlled by the floral homeotic gene AGAMOUS during early organogenesis. Development 132: 429-438.
2. Honma T, Goto K (2000) The Arabidopsis floral homeotic gene PISTILLATA is regulated by discrete cis-elements responsive to induction and maintenance signals. Development 127: 2021-2030.
3. Ito T, Wellmer F, Yu H, Das P, Ito N, et al. (2004) The homeotic protein AGAMOUS controls microsporogenesis by regulation of SPOROCYTELESS. Nature 430: 356-360.
4. Savidge B, Rounsley SD, Yanofsky MF (1995) Temporal relationship between the transcription of two Arabidopsis MADS box genes and the floral organ identity genes. Plant Cell 7: 721-733.
