# Supplementary material for: Genome-Wide Analysis of Gene Expression during Early Arabidopsis Flower Development
Source: PLoS Genet. 2006 Jul 28;2(7):e117. doi: 10.1371/journal.pgen.0020117 (PMC1523247; doi:10.1371/journal.pgen.0020117)
Supplement: Table S9 — Gene identifiers and corresponding primer sequences are shown. (33 KB XLS) [file pgen.0020117.st009.pdf]

Table S9: Primers used to generate probes for *in situ* hybridizations. Gene identifiers and corresponding primer sequences are shown.

Gene	Primer Sequence
At1g12080	5'-GCCTTCTCAACGGCGACTTC-3'
	5'-CCAACAAAGACAGTGGAGGA-3'
At2g35310	5'-CATCACCCAAATATGAAGAAGTCATGG-3'
	5'-GCTACATAGATCCATCCGATAGACATTC-3'
At5g22430	5'-CGCTTTAGCCGTCTTCTCCAACTTG-3'
	5'-GGTGGGAATCCAAAACCTCCTGC-3'
At1g05480	5'-GCCGGTACAATCATGTTGAACGAGC-3'
	5'-CATGCACAAATGGAAGCATAACAGCC-3'
At3g04290	5'-GGAATTGGAATCCTCAACGACACTG-3'
	5'-CCAATAGCATGGCCGTGCTAAG-3'
At3g46770	5'-CCAAATCAAACCGCGGATGACATG-3'
	5'-CCTTGATAATGTGGACCTTGATTTCGTG-3'
At5g66940	5'-GCTACGCCTGTTCTTTTCCCTCAG-3'
	5'-CGCTACGTAGTCTCCAGACACG-3'
At5g57720	5'-CCTCCATATCCAGATTTCCTCAAGATC-3'
	5'-CCGAATCCATCGATTAGATTGACCG-3'
At3g53310	5'-CTGATCCATTGCCAAAGACAGTGAAG-3'
	5'-CCTTCTGCAAATCCTGTCCCATC-3'
At3g26744	5'-CAATGATCTTCACAATGAACTTGA-3'
	5'-CAGCATACCCTGCTGTATCG-3'
At2g04570	5'-GCTTAAACCAATCATTCCGGCGTAC-3'
	5'-GTGCTGTTCATGAGAGCATTGGC-3'
At5g65590	5'-CCTTCGTCTTCTTCCTCTCAGAG-3'
	5'-CTTAAACTTACCAAGATTCAAGGCAATGC-3'
At1g21460	5'-CGTTTTCGGAAATGCAACTGCTCTG-3'
	5'-GCTTCTTCTCATCATCTTTCATCTCCAC-3'
