# Supplementary material for: Genome-Wide Analysis of Gene Expression during Early Arabidopsis Flower Development
Source: PLoS Genet. 2006 Jul 28;2(7):e117. doi: 10.1371/journal.pgen.0020117 (PMC1523247; doi:10.1371/journal.pgen.0020117)
Supplement: Table S11 — The mode of an interaction (direct or indirect) is specified, if known. Abbreviations: AG: AGAMOUS; AGL24: AGAMOUS-LIKE24; ANT: AINTEGUMENTA; AP1: APETALA1; AP2: APETALA2; AP3: APETALA3; ASK1: ARABIDOPSIS SKP-LIKE1; BLR: BELLRINGER; CAL: CAULIFLOWER; CLF: CURLY LEAF; CLV3: CLAVATA3; CRC: CRABS CLAW; FT: FLOWERING LOCUS T; FUL: FRUITFULL; GA4: GA REQUIRING 4; LEU: LEUNIG; LFY: LEAFY; miR172: microRNA172; NAP: NAC-LIKE, ACTIVATED BY AP3/PI; NZZ/SPL: NOZZLE/SPOROCYTELESS; PI: PISTILLATA; RBE: RABBIT EARS; SAP: STERILE APETALA; SEP1–4: SEPALLATA1–4; SEU: SEUSS; SHP2: SHATTERPROOF2; SUP:SUPERMAN; TFL1: TERMINAL FLOWER 1; UFO: UNUSUAL FLORAL ORGANS; WUS: WUSCHEL. (41 KB PDF) [file pgen.0020117.st011.pdf]

Table S11: Selected references for the gene interactions summarized in the network diagram shown in Figure 1. The mode of an interaction (direct or indirect) is specified, if known. Abbreviations: AG: AGAMOUS; AGL24: AGAMOUS-LIKE24; ANT: AINTEGUMENTA; AP1: APETALA1; AP2: APETALA2; AP3: APETALA3; ASK1: ARABIDOPSIS SKP-LIKE1; BLR: BELLRINGER; CAL: CAULIFLOWER; CLF: CURLY LEAF; CLV3: CLAVATA3; CRC: CRABS CLAW; FT: FLOWERING LOCUS T; FUL: FRUITFULL; GA4: GA REQUIRING 4; LEU: LEUNIG; LFY: LEAFY; miR172: microRNA172; NAP: NAC-LIKE, ACTIVATED BY AP3/PI; NZZ/SPL: NOZZLE/SPOROCYTELESS; NUB: NUBBIN; PI: PISTILLATA; RBE: RABBIT EARS; SAP: STERILE APETALA; SEP1-4: SEPALLATA1-4; SEU: SEUSS; SHP2: SHATTERPROOF2; SUP:SUPERMAN; TFL1: TERMINAL FLOWER 1; UFO: UNUSUAL FLORAL ORGANS; WUS: WUSCHEL.

Interaction	Mode	Reference
AG promotes AG	Direct	[1]
AG promotes AP3	Direct	[1]
AG promotes CRC	Direct	[1]
AG promotes GA4	Direct	[1]
AG promotes NUB	Direct	[1]
AG promotes NZZ/SPL	Direct	[2]
AG promotes SEP3	Direct	[1]
AG promotes SHP2		[3]
AG represses AP1		[4]
AG represses WUS		[5,6]
ANT represses AG		[7]
AP1 promotes AP3		[8]
AP1 promotes LFY		[9,10]
AP1 promotes PI		[8]
AP1 represses AGL24		[11]
AP1 represses FUL		[12]
AP1 represses TFL1		[9,13]
AP2 represses AG		[14]
AP3 interacts with PI		[15,16]
AP3/PI promote AP3		[17]
AP3/PI promote NAP		[18]
AP3/PI promote PI	Indirect	[17]
AP3/PI promote SUP		[19]
BLR represses AG		[20]
CAL promotes LFY		[10]
CAL represses TFL1		[9,13]
CLF represses AG		[21]
CLV3 represses WUS		[22,23]
FT interacts with FD		[24,25]
FT/FD promote AP1	Direct	[24,25]
FUL promotes LFY		[12]
LFY promotes AG	Direct	[5,6,26,27,28]

LFY promotes AP1	Direct	[27,29,30]
LFY promotes AP3		[26,27,31]
LFY promotes CAL	Direct	[30]
LFY promotes PI		[26]
LFY promotes SEP1		[32,33]
LFY promotes SEP2		[32,33]
LFY promotes SEP3		[32]
LFY promotes SUP		[19]
LFY represses TFL1		[9,13]
LUG interacts with SEU		[34]
LUG/SEU repress AG		[35,36,37]
miR172 represses AP2		[38]
RBE represses AG		[39]
SAP represses AG		[40]
SUP represses AP3		[41,42]
SUP represses PI		[41,42]
TFL1 represses AP1		[9,13]
TFL1 represses LFY		[9,13]
UFO interacts with ASK1		[43]
UFO/ASK1 promote AP3		[44,45,46]
UFO/ASK1 promote PI		[44,45,46]
WUS promotes AG	Direct	[5,6]
WUS promotes CLV3		[22,23]

References:

1. Gomez-Mena C, de Folter S, Costa MM, Angenent GC, Sablowski R (2005) Transcriptional program controlled by the floral homeotic gene AGAMOUS during early organogenesis. Development 132: 429-438.
2. Ito T, Wellmer F, Yu H, Das P, Ito N, et al. (2004) The homeotic protein AGAMOUS controls microsporogenesis by regulation of SPOROCYTELESS. Nature 430: 356-360.
3. Savidge B, Rounsley SD, Yanofsky MF (1995) Temporal relationship between the transcription of two Arabidopsis MADS box genes and the floral organ identity genes. Plant Cell 7: 721-733.
4. Gustafson-Brown C, Savidge B, Yanofsky MF (1994) Regulation of the arabidopsis floral homeotic gene APETALA1. Cell 76: 131-143.
5. Lenhard M, Bohnert A, Jurgens G, Laux T (2001) Termination of stem cell maintenance in Arabidopsis floral meristems by interactions between WUSCHEL and AGAMOUS. Cell 105: 805-814.
6. Lohmann JU, Hong RL, Hobe M, Busch MA, Parcy F, et al. (2001) A molecular link between stem cell regulation and floral patterning in Arabidopsis. Cell 105: 793-803.
7. Krizek BA, Prost V, Macias A (2000) AINTEGUMENTA promotes petal identity and acts as a negative regulator of AGAMOUS. Plant Cell 12: 1357-1366.
8. Ng M, Yanofsky MF (2001) Activation of the Arabidopsis B class homeotic genes by APETALA1. Plant Cell 13: 739-753.

9. Liljegren SJ, Gustafson-Brown C, Pinyopich A, Ditta GS, Yanofsky MF (1999) Interactions among APETALA1, LEAFY, and TERMINAL FLOWER1 specify meristem fate. Plant Cell 11: 1007-1018.
10. Bowman JL, Alvarez J, Weigel D, Meyerowitz EM, Smyth DR (1993) Control of flower development in Arabidopsis thaliana by APETALA1 and interacting genes. Development 119: 721-743.
11. Yu H, Ito T, Wellmer F, Meyerowitz EM (2004) Repression of AGAMOUS-LIKE 24 is a crucial step in promoting flower development. Nat Genet 36: 157-161.
12. Ferrandiz C, Gu Q, Martienssen R, Yanofsky MF (2000) Redundant regulation of meristem identity and plant architecture by FRUITFULL, APETALA1 and CAULIFLOWER. Development 127: 725-734.
13. Ratcliffe OJ, Bradley DJ, Coen ES (1999) Separation of shoot and floral identity in Arabidopsis. Development 126: 1109-1120.
14. Drews GN, Bowman JL, Meyerowitz EM (1991) Negative regulation of the Arabidopsis homeotic gene AGAMOUS by the APETALA2 product. Cell 65: 991-1002.
15. Riechmann JL, Krizek BA, Meyerowitz EM (1996) Dimerization specificity of Arabidopsis MADS domain homeotic proteins APETALA1, APETALA3, PISTILLATA, and AGAMOUS. Proc Natl Acad Sci U S A 93: 4793-4798.
16. Honma T, Goto K (2001) Complexes of MADS-box proteins are sufficient to convert leaves into floral organs. Nature 409: 525-529.
17. Honma T, Goto K (2000) The Arabidopsis floral homeotic gene PISTILLATA is regulated by discrete cis-elements responsive to induction and maintenance signals. Development 127: 2021-2030.
18. Sablowski RW, Meyerowitz EM (1998) A homolog of NO APICAL MERISTEM is an immediate target of the floral homeotic genes APETALA3/PISTILLATA. Cell 92: 93-103.
19. Sakai H, Krizek BA, Jacobsen SE, Meyerowitz EM (2000) Regulation of SUP expression identifies multiple regulators involved in arabidopsis floral meristem development. Plant Cell 12: 1607-1618.
20. Bao X, Franks RG, Levin JZ, Liu Z (2004) Repression of AGAMOUS by BELLRINGER in floral and inflorescence meristems. Plant Cell 16: 1478-1489.
21. Goodrich J, Puangsomlee P, Martin M, Long D, Meyerowitz EM, et al. (1997) A Polycomb-group gene regulates homeotic gene expression in Arabidopsis. Nature 386: 44-51.
22. Brand U, Fletcher JC, Hobe M, Meyerowitz EM, Simon R (2000) Dependence of stem cell fate in Arabidopsis on a feedback loop regulated by CLV3 activity. Science 289: 617-619.
23. Schoof H, Lenhard M, Haecker A, Mayer KF, Jurgens G, et al. (2000) The stem cell population of Arabidopsis shoot meristems in maintained by a regulatory loop between the CLAVATA and WUSCHEL genes. Cell 100: 635-644.
24. Wigge PA, Kim MC, Jaeger KE, Busch W, Schmid M, et al. (2005) Integration of spatial and temporal information during floral induction in Arabidopsis. Science 309: 1056-1059.

25. Abe M, Kobayashi Y, Yamamoto S, Daimon Y, Yamaguchi A, et al. (2005) FD, a bZIP protein mediating signals from the floral pathway integrator FT at the shoot apex. Science 309: 1052-1056.
26. Weigel D, Meyerowitz EM (1993) Activation of Floral Homeotic Genes in Arabidopsis. Science 261: 1723-1726.
27. Parcy F, Nilsson O, Busch MA, Lee I, Weigel D (1998) A genetic framework for floral patterning. Nature 395: 561-566.
28. Busch MA, Bomblies K, Weigel D (1999) Activation of a floral homeotic gene in Arabidopsis. Science 285: 585-587.
29. Wagner D, Sablowski RW, Meyerowitz EM (1999) Transcriptional activation of APETALA1 by LEAFY. Science 285: 582-584.
30. William DA, Su Y, Smith MR, Lu M, Baldwin DA, et al. (2004) Genomic identification of direct target genes of LEAFY. Proc Natl Acad Sci U S A 101: 1775-1780.
31. Lamb RS, Hill TA, Tan QK, Irish VF (2002) Regulation of APETALA3 floral homeotic gene expression by meristem identity genes. Development 129: 2079-2086.
32. Schmid M, Uhlenhaut NH, Godard F, Demar M, Bressan R, et al. (2003) Dissection of floral induction pathways using global expression analysis. Development 130: 6001-6012.
33. Wagner D, Wellmer F, Dilks K, William D, Smith MR, et al. (2004) Floral induction in tissue culture: a system for the analysis of LEAFY-dependent gene regulation. Plant J 39: 273-282.
34. Sridhar VV, Surendrarao A, Gonzalez D, Conlan RS, Liu Z (2004) Transcriptional repression of target genes by LEUNIG and SEUSS, two interacting regulatory proteins for Arabidopsis flower development. Proc Natl Acad Sci U S A 101: 11494-11499.
35. Franks RG, Wang C, Levin JZ, Liu Z (2002) SEUSS, a member of a novel family of plant regulatory proteins, represses floral homeotic gene expression with LEUNIG. Development 129: 253-263.
36. Liu Z, Meyerowitz EM (1995) LEUNIG regulates AGAMOUS expression in Arabidopsis flowers. Development 121: 975-991.
37. Conner J, Liu Z (2000) LEUNIG, a putative transcriptional corepressor that regulates AGAMOUS expression during flower development. Proc Natl Acad Sci U S A 97: 12902-12907.
38. Chen X (2003) A MicroRNA as a Translational Repressor of APETALA2 in Arabidopsis Flower Development. Science.
39. Krizek BA, Lewis MW, Fletcher JC (2006) RABBIT EARS is a second-whorl repressor of AGAMOUS that maintains spatial boundaries in Arabidopsis flowers. Plant J 45: 369-383.
40. Byzova MV, Franken J, Aarts MG, de Almeida-Engler J, Engler G, et al. (1999) Arabidopsis STERILE APETALA, a multifunctional gene regulating inflorescence, flower, and ovule development. Genes Dev 13: 1002-1014.
41. Bowman JL, Sakai H, Jack T, Weigel D, Mayer U, et al. (1992) SUPERMAN, a regulator of floral homeotic genes in Arabidopsis. Development 114: 599-615.

42. Sakai H, Medrano LJ, Meyerowitz EM (1995) Role of SUPERMAN in maintaining Arabidopsis floral whorl boundaries. Nature 378: 199-203.
43. Wang X, Feng S, Nakayama N, Crosby WL, Irish V, et al. (2003) The COP9 signalosome interacts with SCF UFO and participates in Arabidopsis flower development. Plant Cell 15: 1071-1082.
44. Lee I, Wolfe DS, Nilsson O, Weigel D (1997) A LEAFY co-regulator encoded by UNUSUAL FLORAL ORGANS. Curr Biol 7: 95-104.
45. Levin JZ, Meyerowitz EM (1995) UFO: an Arabidopsis gene involved in both floral meristem and floral organ development. Plant Cell 7: 529-548.
46. Zhao D, Yu Q, Chen M, Ma H (2001) The ASK1 gene regulates B function gene expression in cooperation with UFO and LEAFY in Arabidopsis. Development 128: 2735-2746.
